# Defining shapes of two-dimensional crystals with undefinable edge energies

**DOI:** 10.1038/s43588-022-00347-5

**Published:** 2022-11-28

**Authors:** Luqing Wang, Sharmila N. Shirodkar, Zhuhua Zhang, Boris I. Yakobson

**Affiliations:** 1 Department of Materials Science and NanoEngineering, Rice University, Houston, TX USA; 2 Department of Chemistry, Rice University, Houston, TX USA

**Keywords:** Computational methods, Two-dimensional materials

## Abstract

The equilibrium shape of crystals is a fundamental property of both aesthetic appeal and practical importance: the shape and its facets control the catalytic, light-emitting, sensing, magnetic and plasmonic behaviors. It is also a visible macro-manifestation of the underlying atomic-scale forces and chemical makeup, most conspicuous in two-dimensional (2D) materials of keen current interest. If the crystal surface/edge energy is known for different directions, its shape can be obtained by the geometric Wulff construction, a tenet of crystal physics; however, if symmetry is lacking, the crystal edge energy cannot be defined or calculated and thus its shape becomes elusive, presenting an insurmountable problem for theory. Here we show how one can proceed with auxiliary edge energies towards a constructive prediction, through well-planned computations, of a unique crystal shape. We demonstrate it for challenging materials such as SnSe, which is of *C*_2v_ symmetry, and even AgNO_2_ of *C*_1_, which has no symmetry at all.

## Abstract (web summary)

The indeterminacy of edge or surface energy — unknowable for low-symmetry crystals — is avoided by an algebraic system complemented with closure equations, which enables computing algorithms to predict the equilibrium shape of any crystal.

## Main

We instantly associate the very word crystal with a shape (and perhaps color, or the lack of it), which has often been perfected through slow geological formation or craftsmanship. Physical systems in equilibrium arrive at a state of minimal energy. Crystals—oblivious to this fundamental principle—achieve their shapes by billions of constituent atoms relentlessly performing a trial and error experiment until they reach the equilibrium shape. For us to predict a crystal shape, such an approach is impossible, and so theories usually reduce the search to the exterior (surface or edge) energy minimization only^1,2^, whereas the interior-bulk (volume or area) remains invariant. If the exterior energy density, such as the angle-dependent surface energy *ε*(*a*), is given for all direction angles *a*, this should be sufficient to define the crystal shape, as epitomized by the famed Wulff construction^2–5^—a geometrical recipe derived from surface energy, in which the answer emerges as an envelope of planes or lines that are distanced by *ε*(*a*) from some point and drawn for all directions *a*.

One century later, the advent of two-dimensional (2D) materials^6–9^ made such analysis particularly appealing, helped by a daily growing abundance of shape imagery (it is easier to characterize a 2D rather than a three-dimensional (3D) shape, not to mention improved microscopy). One can learn whether the crystal reached equilibrium or was shaped kinetically, learn about the edge-structures, and the environment. Furthermore, advances in the first-principles-based computations—notably density functional theory, DFT—nicely complete the Wulff construction by offering *ε*(*a*), at the desired accuracy, to predict a crystal’s shape all of the way from its elemental chemical makeup. Such a plan has been successfully realized in numerous cases in which there was a definition for the edge or surface energy. As the primary well-defined quantity is always the total energy *E*_t_, one typically resorts to a ribbon (or slab, in 3D) to define the edge energy (per length) as an excess *ε* = (*E*_t_ – *E*_b_)/2*l* (where *l* is the lattice constant) over the energy of unbounded bulk material *E*_b_. This works if the opposite edges are indistinguishable by symmetry, but fails otherwise, yielding a meaningless average *ε*. In some cases, the approach can be augmented by considering a symmetric polygon or polyhedron with all sides identical, as has been realized for 3D GaAs (ref. ^10^), more recently for 2D hexagonal boron nitride (hBN) (ref. ^11^) and for metal chalcogenides^12^—a broad family^6–8^. This method cannot be taken for granted. Many materials lack sufficient symmetry to design a sample with identical edges (or surfaces). Then, the mere definition of surface energy seems to vanish—a disturbing yet simple reality highlighted by Cahn and colleagues^13,14^ as gauge invariance. Their studies show that certain changes to the angle-dependent surface energy *ε*(*a*) yield an unchanged Wulff shape; hence the latter does not define the surface energy for all directions. A far reaching yet not often appreciated corollary is that the determination of energy for the surface of crystals (of low symmetry) is impossible^13^; the absolute value can never be known in principle^15^. The paradox of the Wulff construction is that it states how to obtain the shape from a given edge energy, but the definition of the latter is left out. Cahn and colleagues went further to show that such a definition is indeed fundamentally absent, but did not offer a solution. Yet we know that nature does find the answer, for each crystal—a true shape. This poses a compelling problem: how to find it in theory?

## Results

### Y- and *y*-crystals as an abstraction of materials

**Fig. 1:**
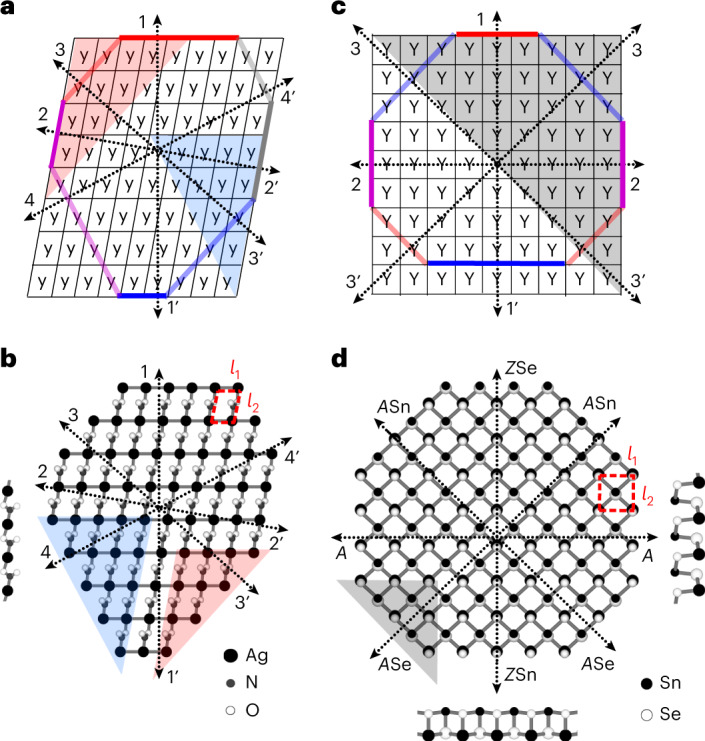
The asymmetric 2D crystals. **a**,**b**, The *C*_1_-symmetry *y*-crystal (**a**) mimics the AgNO_2_ monolayer (**b**) with the same lattice constants *l*_1_ = 3.39, *l*_2_ = 4.93; angle ∠*θ* = 79.5° (ref. ^16^). Arrows are the normals to eight basic edges (thick solid lines), whereas the red and blue shading indicate two non-equivalent triangles. The left inset in **b** is the side view. **c**,**d**, The *C*_2v_-symmetry Y-crystal (**c**) mimics the SnSe monolayer (**d**), with *l*_1_ = 4.22, *l*_2_ = 4.52; ∠*θ* = 90° (ref. ^18^). Thick lines highlight five basic edges, with their normals as arrows. In **d** the small and big atoms distinguish between the top and bottom layers of the SnSe, whereas the right and bottom insets are side views.

A fully asymmetric (*C*_1_) gedanken crystal of *y* vividly illustrates such a challenge (Fig. 1a): no matter how the sample is cut out (ribbon, triangle, circle or any other), it is not surrounded by identical edges. This renders their energies elusive and the equilibrium shape unpredictable by the standard Wulff construction. For a truly 2D planar monoatomic crystal, a necessary and sufficient condition for edge-energy indeterminacy is simply the absence of both inversion *C*_2_ and threefold rotation *C*_3_. To be clear, we show a fully asymmetric monolayer of silver nitrite^16,17^ (Fig. 1b) and a well-studied 2D SnSe^18–24^ (Fig. 1c,d)—the latter is of *C*_2v_ symmetry, which is slightly higher yet insufficient for separating and defining its edge energy. Its sketch-depiction (Y-crystal; Fig. 1c) has an advantage: it is not cluttered with atoms and bonds, and thus clearly displays the SnSe features, which is essential for the compelling problem of finding the shape.

Here we offer a solution, demonstrating that the shape of even the lowest-symmetry *C*_1_ crystal (that is, no-symmetry) can be obtained through well-planned calculations (possibly from an ab initio or, for that matter, any other atomistic model permitting total energy evaluation). In all cases, the directions can be chosen along the Bravais lattice vectors—supplemented by the diagonals (see Supplementary Section 1)—to serve as basic edges; we can then try to obtain ε(*a*) for all directions (basic edges must be reconstructed to the lowest energy for a real material). The total energies of the selected polygons allow one to relate the basic edge energy by linear algebraic equations, which turn out to be underdetermined and require the introduction of arbitrary parameters. Nevertheless, as we see, the shapes obtained in this way remain unchanged (a manifestation of the above-mentioned gauge invariance^13^) by these auxiliary parameters and thus emerge as true equilibrium shapes. We first demonstrate it for *C*_2v_ symmetry (using SnSe) and then for a general no-symmetry *C*_1_ case (with AgNO_2_, for example). We further include the role of chemical potentials for binary and ternary compositions, analyze hBN to test the method and describe the symmetry classification (Supplementary Table 4).

**Fig. 2:**
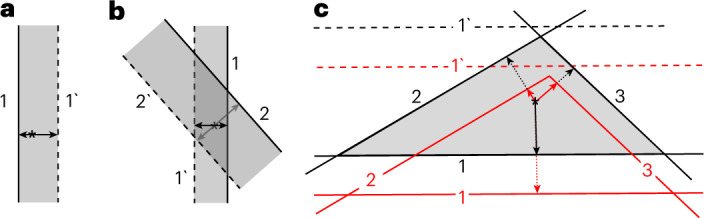
Wulff constructions for hypothetic materials with one, two or three easy-cleavage (low-energy fracture) directions. **a**–**c**, Materials with one, two or three easy-cleavage (low-energy fracture) directions yield a ribbon-strip (**a**), parallelogram (**b**) or a triangle (shaded gray) (**c**), respectively. The arrows—from the center-asterisks to the edges—are the distances equal to the corresponding edge energies *ε*_1_, *ε*_1′_ and so on, as lableled. The red color in **c** marks the construction obtained when the (undefined, auxiliary) edge energy value *ε*_1_ is arbitrarily increased. See Methods for details.

To see how to arrive at this methodology, consider examples of merely heuristic value. Imagine a material with a single easy-cleavage direction, which, in absence of symmetry, would have two different basic edge energies. Its Wulff construction width is fixed by one equation only (*ε*_1_ + *ε*_1′_ = *E*_11′_, that is, the total edge energy of a strip), and is otherwise unconstrained, free to move in plane, with its position undetermined but its shape obviously unchanged (Fig. 2a). For a material with two inherently easy cuts in non-equivalent directions (Fig. 2b), or with three cuts and opposite edges pairs (Fig. 2c), the indeterminacy is 2 for both. One learns that an indeterminacy of 2 is the maximum (any symmetry axis would supply one equation, reducing the indeterminacy to 1, or to 0 for high symmetries).

### Algebraic master system, indeterminacy and closure equations

We begin from *C*_2v_ symmetry materials such as SnS, GeS, GeSe^24^ and many others^16^, which have only two determinable edge energies. An example of great current interest^19–24^ is SnSe, whose orthorhombic cell and buckled hexagonal lattice with parallel grooves (Fig. 1d) resemble the familiar phosphorene^4^, but are distinguished by the off-plane tilt of the Sn–Se bonds. Abstracting from the chemical composition, our Y-crystal is isomorphic (Supplementary Section 3) to SnSe, with both having five non-equivalent basic edges marked by their normals (Fig. 1c), with energies *ε*_1_ and *ε*_1′_, *ε*_2_, *ε*_3_ and *ε*_3′_ (where the prime symbols note the inverse directions so that *ε*_2_ = *ε*_2’_ by symmetry).

Commonly, the basic edge energies are determined (computed) by choosing a sample enclosed by only one edge type: a ribbon for any inversion-symmetric crystal, or an equilateral triangle for a trigonal symmetry such as hBN. This is impossible for the Y-crystal, whose symmetry is insufficient. Apart from *ε*_2_ (for which a ribbon can be constructed; see equation (2)), all other basic edges cannot be singled out by any cutout. Consequently, for five unknowns (basic edge energies), only four independent equations can be set up, using ribbons and triangles (shaded in Fig. 1c) with different edges:

1 $$\varepsilon _1 + \varepsilon _{1\prime } = E_{11\prime }/l_1,$$

2 $$\varepsilon _2 = E_{22}/2l_2,$$

3 $$\varepsilon _3 + \varepsilon _{3\prime } = E_{33\prime }/l_3,$$

4 $$\varepsilon _1l_1 + {{{\mathrm{ }}}}\varepsilon _2l_2 + {{{\mathrm{ }}}}\varepsilon _{3\prime }l_3 = {{{\mathrm{ }}}}E_{123\prime },$$

where the lengths are measured in ångstroms and energies in electron volts; henceforth, we omit these units for brevity (Supplementary Section 4). The right hand side (RHS) values are all well defined, computable total energies of ribbons or triangles (two or three subscripts, respectively) taken relative to the bulk crystal energy, that is, the chemical potential of the Y-component in the 2D-bulk phase (*μ*_Y_). In equation (4), the RHS must be evaluated for larger *N*-cells-wide/tall triangles and then divided by *N*. Any other polygon is reducible to a combination of the ribbons and triangle already picked (123′), thus yielding no more linearly independent equations (Supplementary Section 2b). For Y-crystal illustration, we arbitrarily pick reasonable values (such as 0.14, 0.10, 0.10 and 1.11) for the RHS of equations (1–4). Having five unknowns but only four equations, this system is underdetermined and thus one cannot obtain the basic edge energies, the ε(*a*) or the Wulff construction. We proceed, however, by adding a closure equation, finding the crystal shape and further seeing that the closure equation has no influence on the shape, which therefore is uniquely defined. The closure can be in the form of constraint on any combination of the basic edge energies (for example, *ε*_3_ − *ε*_3’_ = *α*) for an auxiliary; then, at each *α*-value, the system (equations (1–4)) is solved for the basic *ε*_*i*_.

To predict a shape, the choice of basic edges (facets) is always one of the first tasks, and has little to do with the symmetry whether it is high or low. The a priori motivation to choose low-index edges is that, as they are more densely packed, they probably have weak interplane bonding and lower edge energies. Such a choice can be readily augmented by adding any edge, if suggested empirically: it merely increases the rank of equations (1–4), not changing the way in which to overcome the same indeterminacy (Supplementary Section 2c). Furthermore, formally adding *M*-many edges offers a discretization of a continuum *ε*(*a*) function; it costs little in solving ~*M* linear equations, but becomes quite taxing in computing numerous RHS values with DFT. Instead, an economical shortcut seems more practical (even though less rigorous) than discretization with large *M*.

**Fig. 3:**
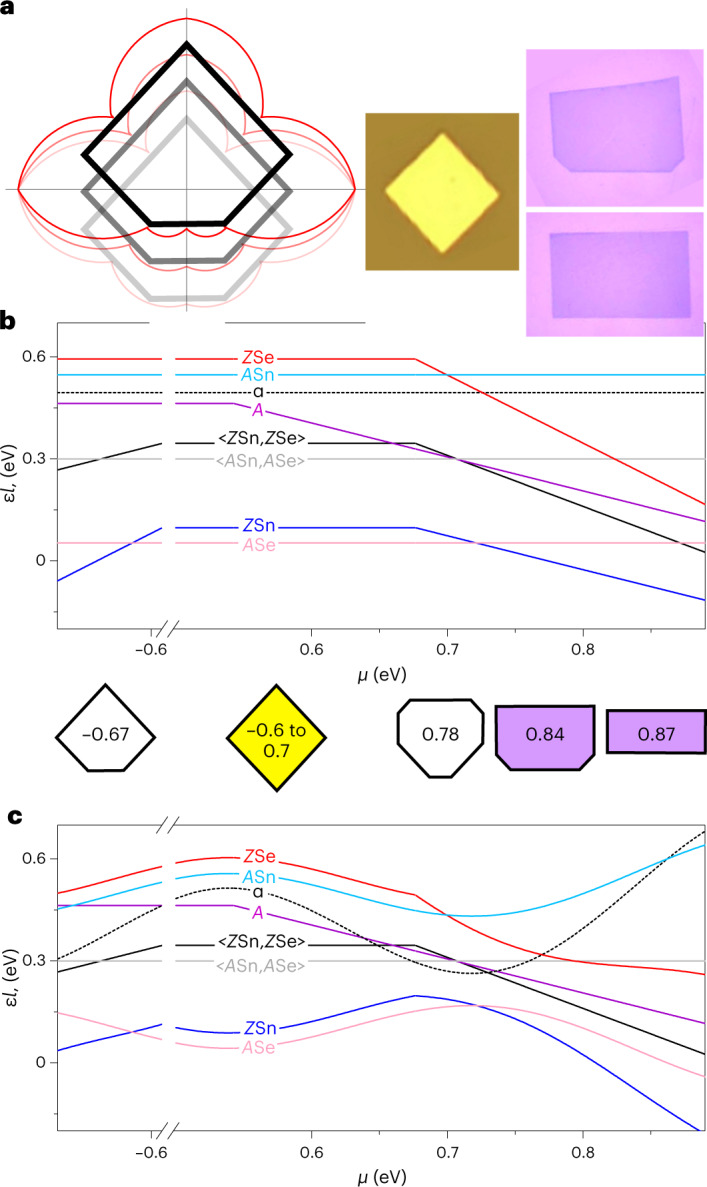
The auxiliary ε-plots and Wulff constructions for the Y-crystal or SnS **a**, The *ε*-plots (red) and Wulff construction shapes (gray) for the Y-crystal at *μ* = −0.67, for *α* ≡ *ε*_3_ − *ε*_3′_ = −0.03, 0 or 0.03. The right insets are experimental images: the yellow rhomb^29^, the violet rectangle^30^ and two-corner truncated rectangle^30^. Panel adapted with permission from: **a**(middle) ref. ^29^, Elsevier; **a**(right) ref. ^30^, IOP. **b**, The auxiliary edge energies of SnSe (at *α* ≡ *ε*_3_ − *ε*_3’_ = 0.08, *l*_3_ = 6.19 Å; the plotted value is *αl*_3_ = 0.49) varying with chemical potential. The corresponding equilibrium polygons, marked by *μ*-values—where yellow rhomb is for −0.6 to 0.7, violet two-corner truncated rectangle is for 0.84 and violet rectangle is for 0.87—are color-shaded to match experimental photos (Supplementary Section 7) in the inset above **b**. **c**, The auxiliary edge energy chosen at random as *αl* = 0.62*μ* + 0.19 sin 15*μ*. Source data

To have a full function *ε*(*a*) at arbitrary *a*, we invoke an ansatz that any slanted, vicinal edge is a sequence of segments projections of the basic edges, and thus its energy is a sum of the basic edge energies, in proper proportions^25–27^, such as *c*_1_*ε*_1_ + *c*_3_*ε*_3_ and so on. Simple trigonometry then results in^25^ the interpolation ansatz: *ε*(*a*) = *ε*|cos(*a* + C)|, with amplitude *ε* and phase C fully defined by the lattice geometry and the basic edge energies (see Supplementary Section 5). With all of the values of *ε*_*i*_ found above, for any *α*, the interpolation ansatz gives the complete energy, *ε*^*α*^(*a*), and the shape of Y-crystal as the Wulff plot (Fig. 3a). Remarkably, the tangent lines envelope of the Wulff plot merely translates with *α* or otherwise gives an unchanged (obeying the gauge invariance^13^) well-defined shape. Note that only *ε*_2_ is physically defined and *α*-independent due to mirror symmetry. All of the other edge energies vary broadly following auxiliary *α*, having no impact on the shape of the crystal. Regarding the convenient interpolation ansatz, it is reassuring that the minima of piecewise interpolation ansatz function *ε*(*a*)—essential to the Wulff plot—all correspond to the basic edges; any refinements to the remote petals of the ε(*a*) in Fig. 3a would not affect the results, that is, the shape that is found is robust to possible interpolation ansatz imprecision. However, for other cases, the number of equations (1–4), their specifics and the closure may vary, they follow the same structure, which can be called the master system (parameters in Supplementary Section 6).

### Auxiliaries versus chemical potentials in real materials

Turning to a factual SnSe, one must account for its binary composition. Its five basic edge-directions copy the Y-crystal, but now some edges are non-neutral, with a specific frontier element (such as the zigzag edge of hBN, which can have either boron or nitrogen) accordingly labeled: at 0° (*ε*_1_) the edge is zigzag with selenium *Z*Se; at 46° (*ε*_3_) the edge is armchair with tin *A*Sn; at 90° (*ε*_2_) the edge is neutral *A*; at 134° (*ε*_3′_) (an inversion of *ε*_3_) the edge is an *A*Se; and at 180° (*ε*_1′_, an inversion of ε_1_) the edge is a *Z*Sn.

The function *ε*(*a*) for SnSe has the same interpolation ansatz form as for the Y-crystal. Its basic edge energies satisfy the master system of equations (1–4). The RHS energies can again be taken relative to the bulk crystal energy *μ*_SnSe_ = *μ*_Se_ + *μ*_Sn_, which is a constant similar to *μ*_Y_ (at moderate temperatures^28^). The elemental chemical potentials depend on physical conditions, bringing about a new variable, the imbalance of chemical potential *μ* ≡ ½(*μ*_Se_ − *μ*_Sn_), whose range is limited by the elemental phase’s precipitation thermodynamics^28^ as well as nucleation barriers. Accordingly, for the triangle 123′ with extra selenium around the perimeter, we must include −*μ* on the RHS of equation (4). For now-specific materials, the RHS values of equations (1-4) are obtained from DFT computations (Supplementary Section 6) of the respective ribbons *E*_11′_/*l*_1_ = 0.47*μ* + 0.44, *E*_22_/2*l*_2_ = 0.10, *E*_33′_/*l*_3_ = 0.10 and the triangle *E*_123′_ = 1.11 in equation (4). At given conditions (for instance, *μ* = −0.67) we again complement the algebraic master system with a closure *ε*_3_ − *ε*_3′_ ≡ *ε*_*A*Sn_ − *ε*_*A*Se_ = *α* and compute the shape (Fig. 3a). As we already learned with the Y-crystal, the shape stays well defined at a given *μ*. To reiterate, although the energy *ε*_2_ ≡ *ε*_A_ = 0.10 is certain, all of the others depend on the auxiliary *α*, which floats freely with no effect on the observable shape. By contrast, *μ* can really impact the shape. Tracking this is straightforward: for any value of *μ*, assume an arbitrary *α*, find the edge energies versus *μ* (plotted in Fig. 3b) and then the shapes. Not the individual edge energies, but only some combinations are definite, such as *ε*_*Z*Se_ + *ε*_*Z*Sn_, *ε*_*A*Se_ + *ε*_*A*Sn_ (thick lines), and *l*_1_*ε*_*Z*Se_ + *l*_3_*ε*_*A*Se_ varying with *μ* at integer slopes. Individual edge energies, however, vary with *α*, whose choice is arbitrary at each *µ*, so the functions *ε*(*μ*) are merely illustrative (thin lines). To emphasize this, Fig. 3c shows how unfixed the edge energies are due to the auxiliary energy *α* chosen as *α*(*μ*)*l*_3_ = 0.62*μ* + 0.19 sin 15*μ*, yet the shapes derived from both plots (between Fig. 3b,c) are definite. For −0.61 < *μ* < 0.70, a rhombus enclosed by *A*Sn and *A*Se edges agrees with observed synthetic SnSe islands^29^. As *µ* increases, the shape becomes a rectangle truncated at two corners, as has also been seen experimentally^30^ (see the insets to the right of Fig. 3a). Together, such facts (and SnS; Supplementary Section 8) corroborate the auxiliary edge energy approach to predicting the equilibrium shapes of low-symmetry crystals.

**Fig. 4:**
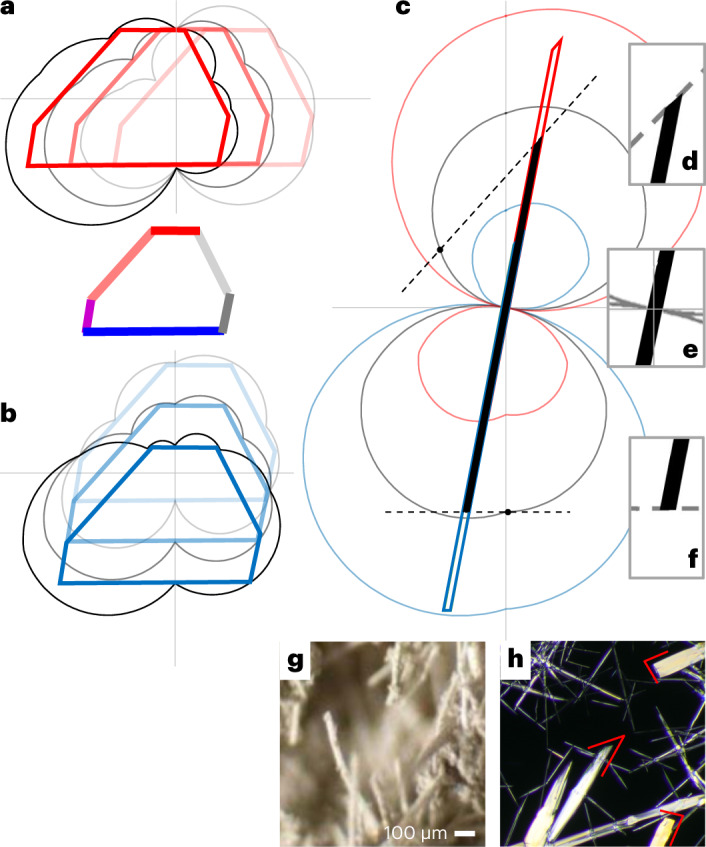
The auxiliary ε-plots and Wulff constructions for *y-*crystal and AgNO_2_. **a**,**b,** For the *y-*crystal, (*α*, *α*′) ≡ (ε_1_ − ε_1′_, ε_2_ − ε_2′_) = (0, 0.3), (0, 0), (0, −0.3) (**a**) and (*α*, *α*′) = (−0.3, 0), (0, 0), (0.3, 0) (**b**). Black lines are the ε-plots, red and blue lines are the Wulff shapes, and the inset shows the invariant Wulff shape with edge colors as in Fig. 1a. **c**, The ε-plot and Wulff constructions for AgNO_2_ at *μ*_Ag_ = *μ*_Ag-bulk_ with blue, gray and red lines for (*α*, *α*′) = (−0.42, 0), (−0.02, 0) and (0.38, 0), respectively. **d**–**f**, Magnifications of **c**. **g**,**h**, Experimental images^31^ of synthesized AgNO_2_, confirming its needle-like structure as computed. The thin red lines highlight the angles at the sample-needle tips in **h**, matching well with those computed in **d** and **f**. Panel **g** adapted from ref. ^31^, Politechnica University of Bucharest. Credit: **h**, Wikipedia. Source data

Now we turn to the most intriguing—not symmetric at all (*C*_1_)—*y*-crystal (Fig. 1a). The eight basic edges are marked by the normals, with energies *ε*_*i*_ (where *i* = 1–4, with primes noting the inverse directions), and where general ε(*a*) has the same interpolation ansatz form (see Supplementary Table 2). In absence of symmetry, the master system extends relative to equations (1–4). Now for the eight unknowns *ε*_*i*_ there are six relations: four with the RHS energies (*E*_*ii*’_) of the ribbons along all basic directions, plus two with the RHS energies *E*_*ijk*_ of the triangles shaded in Fig. 1a (Supplementary equation 3). For the abstract *y*-crystal, one simply picks RHS values in the master system, for instance, 0.5, 0.7, 0.6, 0.8 for the ribbons (*ii*′) and 5.1, 5.4 for the triangles (*ijk*). To be solvable, an underdetermined system must be complemented by two closure conditions, for example, by assigning arbitrary values (*α*, *α*′) to two of the eight indeterminate edges or their combinations. After solving it for the basic edge energies, the interpolation ansatz gives ε^*α*,*α*′^(*a*) for all directions, to produce the shape of the *y*-crystal using the Wulff plots (Fig. 4a,b). Although the *ε-*plots vary with auxiliary energies (*α*, *α*′), the shape only shifts, remaining the same (see inset). This confirms the validity of the auxiliary edge energy method for the no-symmetry (*C*_1_) case, even with an increased number of auxiliaries (2, which is also the maximum for 2D).

A no-symmetry (*C*_1_) material example—a monolayer of silver nitrite AgNO_2_ salt^16,17^ with a triclinic unit cell—can be viewed as a silver lattice with NO_2_ groups inserted between the silver atoms of the sparse direction of *l*_2_ (Fig. 1b). The normals of all eight basic edges are at *a* = 0°, 48.5°, 79.5°, 117.2°, 180°, 228.5°, 259.5° and 297.2°. For AgNO_2_, the use of energy expression ε(*a*) for the arbitrarily oriented edge, as well as the master system relating the eight basic edge energies, are all like the *y*-crystal above. What is new for an actual material is that the RHS values in the master system can now be provided as DFT-computed values: 0.82, 0.01, 0.52, 0.64 for the ribbons, and near 3.15 for the triangles. The tri-elemental composition can still be treated as bi-elemental Ag and NO_2_. With $\mu _{\mathrm{Ag}} + \mu _{\mathrm{NO}_{2}} = \mu _{\mathrm{AgNO}_{2}}$ being invariant, only one physical parameter must be specified, for example, the chemical potential of silver, *μ*_Ag_. It enters the RHS of the master system (Supplementary equation 4) in the following ways (as seen by inspection of Fig. 1b). The *μ*_Ag_ is subtracted from *E*_11’_, *E*_33’_ and *E*_44’_ for the ribbons naturally containing extra silver, but not from *E*_22’_. Similarly, for the triangles (shaded in Fig. 1b) we subtract *μ*_Ag_ from both *E*_123′_ and *E*_12’4_ to account for the extra silver atom per primitive cell.

At a given *μ*_Ag_, for some conditions, the master system requires a closure with two auxiliaries (for example, *α* = *ε*_1_ − *ε*_1′_ and *α*′ = *ε*_2_ − *ε*_2′_) to solve the now-complete master system of eight equations, to determine all *ε*_*i*_ and the entire edge energy function ε(*a*). We do not explore here how *μ*_Ag_ affects the crystal shape (this aspect was already covered for SnSe), but assign its value to the bulk silver, and proceed to predict the shape by solving the master system and finding the Wulff plots. This reveals a shape that is extreme and surprising at first (Fig. 4c). We were able to find confirmation in rather scarce AgNO_2_ experimental evidence^31^ (Fig. 4g,h), in which the crystal shapes are fairly irregular yet strikingly resemble what theory predicts: a highly elongated needle—of no symmetry at all—with one end slanted while another is nearly straight.

### Ranking the definability

Having now shown that the equilibrium crystal shape can be exactly predicted, even for a low-symmetry crystal with an undefinable edge energy, it is useful to briefly rank all 2D materials in this regard. The most common is (1) the trivial-definable case, that is, when inversion symmetry allows for all edge energies to be obtained directly from the total energies of sample-ribbons (for example graphene, phosphorene, SnS_2_). If this is not possible (2), a less obvious regular polygon cutout can be found, and thus we call this case non-trivial-definable: all edge energies can be unambiguously computed and the crystal shape predicted (for example, hBN, MoS_2_, GaS). There are then two levels among undefinable edge energies: (3) when only a pair of opposite edges permit direct definition while all others remain undefinable (as with SnSe, SnS, GeSe, GeS); and (4) the limit of having no symmetry at all as a foothold (as with AgNO_2_) when none of the edge energies give way to definition. In the last two situations, the shape of the crystal can still be theoretically predicted without resorting to empirical data by using the auxiliary edge energy approach (see Supplementary Table 3). An additional test (Supplementary Section 10), with a crystal type such as hBN (2), is to predict its shape through the auxiliary edge energy protocol as if unaware of the existing solution based on equilateral triangles; we arrive at identical results.

## Discussion

Predictions or explanations of equilibrium crystal shapes—traditionally performed through geometrical Wulff construction—relied on the known energy of the surfaces or, in the case of intensely researched 2D materials, their edges. However, for materials of low symmetry, the edge energy cannot be computed or even conceptually defined, and thus one seemingly cannot foresee the shapes without invoking empirical data from experiments^15^. Through a well-planned set of total energy computations, augmented by a concept of auxiliary energies, we demonstrated how one can restore the utility of the Wulff construction and accurately predict the equilibrium shapes of any material. This allowed us to easily include the role of the chemical potential, to explore materials such as SnSe and fully asymmetric AgNO_2_, and to predict their shapes (in accordance with observations).

It is straightforward to generalize this method for 3D crystals, where our master system would grow up to 23 linear algebraic equations (Supplementary Section 11), plus the three constraints with auxiliary parameters needed, still easily solvable for predicting their shapes from first principles. We note a singular attempt^32^ for 3D wurtzite shape, was insightful in considering combinations of surfaces, although relied on experimentally known facets.

At finite temperatures, replacing the RHS of our master system and the ε(*a*) with Gibbs free energies, that is adding entropy terms to DFT-based values, would account for crystal roughening (and round its vertices), well studied and not interfering with our approach. Extended capability to predict shapes of arbitrary crystals is important due to the numerous properties that shapes and edges control in catalysis, light-emission, electronics, sensing, magnetism, plasmonics and so on. The presence of a substrate^33^ reduces the symmetry of 2D-layers; solvent and ligands can be included in calculations, further expanding the utility of the proposed method. Furthermore, recent interest in shift-twisted bilayers, with often low joint symmetry, makes their equilibrated shape a tantalizing target. Crystals of low-symmetry proteins and biomolecules^34^ also offer broad application to understanding their morphology, which is beyond the scope of this work but certainly intriguing. The strategy described above provides a foundation for computational materials science approach to solving the broad range of crystal shape prediction problems that were previously unmanageable.

## Methods

### Methodology of the crystal shape prediction

To arrive at our constructive methodology one should first be clear about the fundamental lack of the surface energy definition. First alluded to in one example^14^ and soon proven to originate from general gauge invariance^13^, undefinable energy still goes against one’s intuition. For uninitiated readers—or, in Cahn’s own terms^15^, "those that are uncomfortable with this"—it is helpful to begin from basic examples. First, imagine a material with a single easy-cleavage direction, which (in absence of symmetry) would have two different basic edge energies. Its Wulff construction width is fixed by one equation only (ε_1_ + ε_1′_ = *E*_11′_, total edge energy of a strip), and is otherwise unconstrained, free to move in plane, with its position undetermined but its shape obviously unchanged (Fig. 2a). Second, if there are two inherently easy cuts in non-equivalent directions, the edge energy equations *ε*_1_ + *ε*_1′_ = *E*_11′_ and *ε*_2_ + *ε*_2′_ = *E*_22′_ leave the indeterminacy as 4 – 2 = 2, permitting both ribbons’ translations but preserving their overlap-parallelogram shape (the Wulff construction; Fig. 2b). Third, with three cuts and opposite edge pairs (Fig. 2c), one, accordingly, has six unknown edge energies, and three (for ribbons 11′, 22′, 33′) plus one (for triangle 123) to make a total of four equations; the indeterminacy is thus 6 – 4 = 2 again; the Wulff construction remains a triangle that can shift in plane without its corners truncated, an invariant shape (Supplementary Section 2a). This hints that, to deal with undefined energies, one can simply formulate all available relations (based on total energies of independent polygons) and repair the indeterminacy by adding any closure equations, which is the strategy we follow. One further learns from these examples that every extra cut adds 2 unknown edge energies but also exactly two non-trivial equations: one for a newly added ribbon and one for a new triangle, and thus the indeterminacy of 2 remains unchanged. Any symmetry axis would supply one equation, reducing the indeterminacy to 1, or to 0 for high symmetries.

### Algorithm for shape determination

In terms of practical steps for the determination of equilibrium shapes for 2D materials, our approach is illustrated as a work-flow chart (Supplementary Fig. 12) and summarized as follows. For any 2D material, one should first judge whether it has undefinable edge energies by its symmetry space group (in practice, it is usually apparent from simply eyeballing the crystal lattice). If it does, examine and determine the basic edges, including the lowest energy reconstructions for each. Second, list an underdetermined set of equations for ribbons and triangles, and perform DFT calculations for the RHS values (DFT calculations of different levels, or even classical empirical potentials of sufficient accuracy, such as ReaxFF, for some elements are equally suitable, depending on the precision versus cost tradeoffs). Third, complement this underdetermined set by the (one or two, as needed) closure equations, and choose and fix the values of the auxiliary energies, solving the equation set to obtain basic edge energies. From which the complete edge energy as a function ε(*a*) of direction-angle *a* can be obtained by the interpolation ansatz equation. Once this is known, one can perform conventional Wulff construction to determine the shape.

It is rather convenient to use the interpolation ansatz as an elegant shortcut, but is not unavoidable: one may prefer instead to merely increase the number of edges to sufficiently many (M), enough to achieve an accurate discretization for a continuous function ε(*a*), for the cost of increased rank of the master system. See the important corollary in Supplementary Section 2c.

### DFT parameters

To obtain numerical values for specific materials, as the RHS of the master system, such as in equations (1-4), the first-principles DFT calculations and structural optimization were performed using the Vienna ab initio simulation package (VASP v.5.4.4)^35^, adopting generalized gradient approximation with the Perdew–Burke–Ernzerhof (PBE)^36^ exchange-correlation functional along with the projector-augmented wave (PAW) potentials. The pseudopotential versions for each element are PAW_PBE tin (08Apr2002), sulfur (17Jan2003), selenium (06Sep2000), silver (06Sep2000), nitrogen (08Apr2002), oxygen (08Apr2002) and boron (06Sep2000). Electronic wave functions were expanded in a plane wave basis set with a kinetic energy cutoff of 400 eV, and, for the Brillouin zone integration, a 9 × 1 × 1 Monkhorst-pack *k*-point mesh was used for ribbon structures. The energy convergence criterion for electronic wave function was set to be 10^−5^ eV. A vacuum layer of about 10 Å in *z*-direction was chosen to guarantee negligible spurious interaction between layers in monolayer simulations using periodic boundary conditions.

### Reporting summary

Further information on research design is available in the Nature Research Reporting Summary linked to this article.

### Supplementary information

Supplementary information Supplementary Sections 1–11. Figs. 1–12 and Tables 1–4.

Reporting Summary

Supplementary Data 1 Atomic structures of Fig 1b in the CIF format.

Supplementary Data 2 Atomic structures of Fig. 1d in the CIF format.

### Source data

Source Data Fig. 3 Source Data for plots.

Source Data Fig. 4 Source Data for plots.

## Data Availability

The DFT data that support the findings of this study are available in Zenodo^37^. Source Data are provided with this paper.

## Code availability

The MATLAB codes used for Wulff plots are available in Zenodo^37^.
