# Publisher Correction: Defining shapes of two-dimensional crystals with undefinable edge energies

**DOI:** 10.1038/s43588-022-00385-z

**Published:** 2022-12-07

**Authors:** Luqing Wang, Sharmila N. Shirodkar, Zhuhua Zhang, Boris I. Yakobson

**Affiliations:** 1 Department of Materials Science and NanoEngineering, Rice University, Houston, TX USA; 2 Department of Chemistry, Rice University, Houston, TX USA

**Keywords:** Computational methods, Two-dimensional materials

Correction to: *Nature Computational Science* 10.1038/s43588-022-00347-5, published online 28 November 2022.

In the version of this article initially published, owing to an error during the production process, the reference citations currently numbered 31 (Fig. 4 caption) and 32–37 (in text) were incorrect. The citations have been corrected in the HTML and PDF versions of the article.

