# Supplementary material for: Defining shapes of two-dimensional crystals with undefinable edge energies
Source: Nat Comput Sci. 2022 Nov 28;2(11):729–35. doi: 10.1038/s43588-022-00347-5 (PMC10766541; doi:10.1038/s43588-022-00347-5)
Supplement: Supplementary file 1 — Supplementary Sections 1–11. Figs. 1–12 and Tables 1–4. [file 43588_2022_347_MOESM1_ESM.pdf]

nature computational science

Article

https://doi.org/10.1038/s43588-022-00347-5

Defining shapes of two-dimensional crystals with undefinable edge energies

In the format provided by the authors and unedited

Table of contents:

Supplementary Section-1. Basic edges.
Supplementary Section-2. Useful geometrical considerations (a-c) and one corollary.
Supplementary Section-3. Symbol correspondence between schematic and real crystals.
Supplementary Section-4. Lattice constants of the crystals.
Supplementary Section-5. Edge energy ε(*a*) interpolation ansatz (IA).
Supplementary Table-1. The parameters of ε(*a*) expression for Y-crystal (and SnSe, SnS).
Supplementary Table-2. The parameters of ε(*a*) expression for *y*-crystal (and $AgNO_2$).
Supplementary Section-6. Master system (MS), its RHS parameters and example edge structures.
Supplementary Section-7. Details to accompany Fig. 3 in the main text.
Supplementary Section-8. SnS Wulff shapes.
Supplementary Section-9. Symmetry classification.
Supplementary Table 3. The types of 2D materials according to the determinacy of ε.
Supplementary Table 4. The classification of 230 space groups according to the determinacy of ε.
Supplementary Section-10. BN Wulff shapes.
Supplementary Section-11. The 23 equations for 3D crystals.
Supplementary Figure 12.
References (*38-46*).

Supplementary Section-1. Basic edges.

We select our basic edges as the primitive lattice vectors $(\bar{l}_1, \bar{l}_2)$, $(-\bar{l}_1, -\bar{l}_2)$ and the diagonals $(\bar{l}_1 + \bar{l}_2)$, $-(\bar{l}_1 + \bar{l}_2)$ and $(\bar{l}_1 - \bar{l}_2)$, $-(\bar{l}_1 - \bar{l}_2)$. Depending on the symmetry of the material, some of the basic edges become identical and hence their contributions to the edge energy (EE). Though this choice may seem *ad hoc*, we show that chemistry of the material dictates this choice.

Consider an arbitrary primitive cell with one orange atom in its basis, as shown in Supplementary Figure 1a. The distance between atoms along l_1 and l_2 is smaller than along the diagonals, and hence the contribution to the edge energy due to bonding along these directions is more pronounced. Hence, in this case only the basic edges $(\bar{l}_1, \bar{l}_2)$, $(-\bar{l}_1, -\bar{l}_2)$ suffice.

On the other hand, on adding an additional basis atom (blue) the bonding along diagonals also becomes important and gives rise to significant contribution to the edge energy. Hence including the diagonals $(\bar{l}_1 + \bar{l}_2)$, $-(\bar{l}_1 + \bar{l}_2)$ and $(\bar{l}_1 - \bar{l}_2)$, $-(\bar{l}_1 - \bar{l}_2)$ is necessary for the estimation of edge energies.

For example, in the case of graphene[25] the two basic edges along the primitive lattice vectors are zigzag edges, as seen below in Supplementary Figure 1d. However, the armchair edge which is along the diagonal has a completely different charge density distribution due to the enhanced bonding between adjacent atoms along this edge. Hence demonstrating that it is important to include the diagonals among the basic edges. Therefore, emphasizing that the EEs of arbitrary directions need both, zigzag (along primitive cell vectors) and armchair (along diagonal direction) edges, to enable the energy decomposition ansatz, we call interpolation ansatz (IA, Supplementary Section-5).

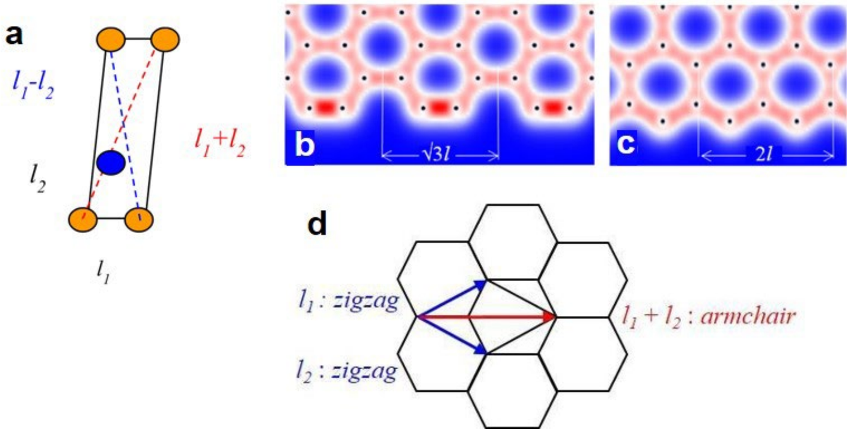

Supplementary Figure 1. (a) An arbitrary primitive cell. Charge density of **(b)** armchair edge and **(c)** zigzag edge in graphene[25]. The armchair edge forms stronger and shorter triple bonds. **(d)** Primitive cell of graphene with zigzag edges along primitive lattice vectors and armchair along the diagonal.

Supplementary Section-2. Useful geometrical considerations (a-c) and one corollary

Supplementary Section-2a Basic case-studies towards methodology: indeterminacy and invariance w.r.t. auxiliaries α's

It is instructive to track the origins of indeterminacy in the master system (MS) equations, through most basic examples. The simplest case is a cut along the *single* preferred-direction, low fracture-energy or easy-cleavage plane, creating two edges. In absence of any symmetry (C_1) they have different energies ε_1 and $\varepsilon_{1`}$ related with well-defined exterior (total minus bulk) 11`-ribbon energy, as

$$\varepsilon_1 + \varepsilon_{1`} = E_{11`},$$

This is all, leaving their absolute values indeterminate, unless a closure equation is added, for instance $\varepsilon_1 - \varepsilon_{1`} = \alpha$, or simply $\varepsilon_1 = \alpha$. Then the Wulff construction is trivial: one line at distance $\varepsilon_1$ to the left (angle $a$ = 180°), another line at $\varepsilon_{1`}$ to the right (a = 0°), yields a strip, Fig. 2a in main text. Importantly, only its width

is fixed by well defined (and computable) physical quantity $E_{11`}$, but absolute positions of the strip—longitudinal and transverse (depending on value α) shifts, are both arbitrary. So, indeterminacy is 2 and the auxiliary parameter only translates the Wulff shape, *without changing its form* (trivial in this case).

Next, *two* cuts in different directions, is a more realistic case, since this allows one to cut out a finite material piece. Then 4 unknown edge-energies ε_1, $\varepsilon_{1`}$ and $\varepsilon_2$, $\varepsilon_{2`}$ satisfy 2 equations only

$\varepsilon_1 + \varepsilon_{1`} = E_{11`}$, and $\varepsilon_2 + \varepsilon_{2`} = E_{22`}$,

leaving again edge-energies indeterminate, in need of two closure equations, e.g.

$\varepsilon_1 - \varepsilon_{1`} = \alpha$, $\varepsilon_2 - \varepsilon_{2`} = \alpha`$.

Morphology from the Wulff construction now is a geometrical parallelepiped (Fig. 2b in main text an overlap of two stripes of widths $E_{11`}$ and $E_{22`}$, whose positions shift with α and α` values. The indeterminacy is again 2, yielding 2 degrees of freedom in the construction translations, while its *shape remains unaffected*.

Third, and sufficiently general to consider, is a material piece enveloped by the *three* cuts (1, 2 and 3) of infinite crystal. Total there are 6 unknown energies: ε_1 and $\varepsilon_{1`}$, $\varepsilon_2$ and $\varepsilon_{2`}$, ε_3 and $\varepsilon_{3`}$. There are 3 equations from the ribbons,

$\varepsilon_1 + \varepsilon_{1`} = E_{11`}$, $\varepsilon_2 + \varepsilon_{2`} = E_{22`}$, $\varepsilon_3 + \varepsilon_{3`} = E_{33`}$,

and there is also a triangle with well defined energy, so

$\varepsilon_1 l_1 + \varepsilon_2 l_2 + \varepsilon_3 l_3 = E_{123}$,

that is 4 equations for 6 unknowns, requiring 6 - 4 =2 closure equations; the indeterminacy being 2. In this case closure can be any pair of linear equations (or nonlinear but one better avoids complications), e. g. $\varepsilon_1 - \varepsilon_{1`} = \alpha$, and perhaps $\varepsilon_1 + \varepsilon_2 - \varepsilon_3 = \alpha`$... Cannot be less than 2 (still underdetermined) and cannot be more than 2 making the system overdetermined, cannot be solved. So, again 2 degrees of freedom.

Further, one can prove by inspection that the arbitrary values of these auxiliaries do not change the Wulff construction shape but only its position. For this, at some choice of α, α`, consider a triangle with edges 1, 2, and 3, each at a distance $\varepsilon_1$, $\varepsilon_2$ and $\varepsilon_3$ from the center (an asterisk in the figure), according to the W. c. rules. In principle, changing α, α` affects all ε's values, shifting the position of the edge-lines and possibly truncating the triangle at the corners, turning Wulff construction into irregular quadra-, penta- or hexagon. Assuming for simplicity the opposite edge-lines 2` and 3` to be far enough not to participate, we focus on 1 and 1` shift caused by some increase of auxiliary α. Distance between them being fixed, increase of α makes $\varepsilon_1$ greater, shifting the strip 11` down, to the red horizontal lines in the figure, and possibly truncates the initial triangle at its top. However, greater ε_1, must also change ε_2 and ε_3 to generally smaller values, to keep the sum $\varepsilon_1 l_1 + \varepsilon_2 l_2 + \varepsilon_3 l_3$ constant, and thus shifting lines 2 and 3 to new positions, forming a new triangle (red in the drawing **c** above). The red triangle must have the same area, as a sum of three bases ε_i times heights l_i, line above, and therefore the same total vertical height as the original black triangle. The latter is important, indicating that the top vertex lies *under* the new 1`-edge (red dashed line), so the triangle is *not* truncated into trapezia. The change of auxiliary parameter only *translates the Wulff construction without changing shape* (here, black to red triangle), Q.E.D.

Examples above show that although auxiliary values do affect the edge energy densities, the Wulf construction shape remains unchanged, except translation. With increasing number of sides-edges, the complexity of drawings rises, but rendering the Wulff construction from the edge-energies can be coded, and this is what Figs. 3 and 4 on main text represent: computer-assisted *proof by construction*, common in geometry. In presence of a symmetry axis (as in C_2) obviously the translation is only possible along the axis, as in main text Fig. 3, while in no-symmetry C_1 case it occurs in two directions, as in Fig. 4.

Supplementary Section-2b Polygons redundancy

One can imagine many polygons based on set of basic edges, This makes obtaining more equations to resolve the indeterminacy and define the edge energies tempting, but one learns that it does not work, since any added polygon (within the same chosen set of edges/cuts) yields no nontrivial equations, as discussed below.

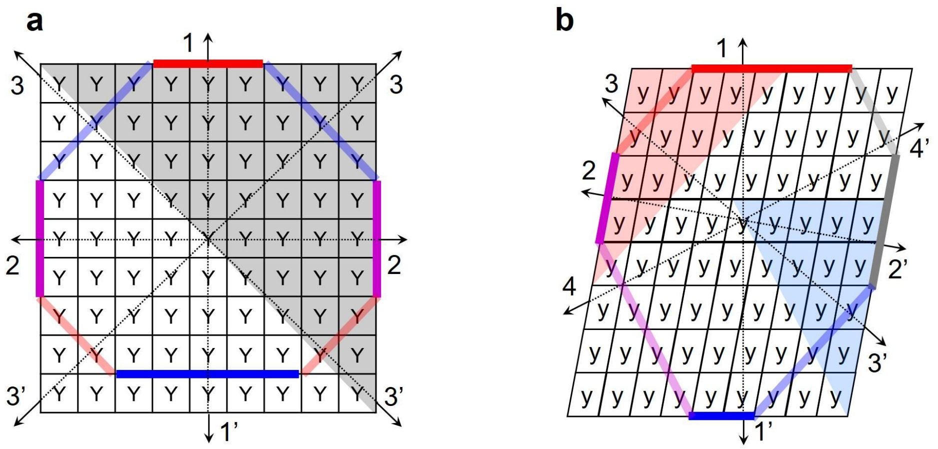

Supplementary Figure 2. (a) Y-crystal and **(b)** *y*-crystal geometry, with gray shaded triangles.

In Supplementary Figure 2a, the edge energy of gray shaded triangle is

$$E_{grey} = \varepsilon_1 + \varepsilon_2 + \varepsilon_{3'}.$$

If we add-join the remaining white inverted-triangle to the gray triangle, thus eliminating the edges 3 and 3', then the edge energy of combined gray-white rectangle is:

$$E_{grey+white} = (\varepsilon_1 + \varepsilon_2 + \varepsilon_{3'}) + (\varepsilon_{1'} + \varepsilon_2 + \varepsilon_3) - (\varepsilon_{3'} + \varepsilon_3)$$
$$= (\varepsilon_1 + \varepsilon_{1'}) + (\varepsilon_2 + \varepsilon_2) = E_{11'}+E_{22},$$

that is reduced to a combination of 2 ribbons edge energy. Therefore, the new (white) triangle brings no new independent equation. In other words, besides the basic-edge ribbons and the selected (grey) triangle (for "Y" crystal, there're 3 ribbons and 1 triangle), adding other polygons only results in repeating those basic-edge ribbons and single triangle equations, and won't introduce new information.

It is easy to sketch (for brevity) a general proof by mathematical induction. Let's assume that for an N-gon it is true that all perimeter edge energy is a linear combination of the energies of the three basic ribbons and one basic triangle. Then attaching one more basic triangle to it builds a new (N+1)-gon, whose energy obviously consists of N-gon plus the added triangle minus a ribbon (due to eliminated edges at the contact). Therefore, the (N+1)-gon energy is also a linear combination of the energies of the three basic ribbons and one basic triangle. And so on, by induction, any polygon adds no new independent equations, Q.E.D. Alternatively, instead of incremental attachment of basic triangles, any picked "new" polygon can be tessellated into basic triangles (plus/minus energies of the contacts-cuts), to see that *such new polygon is redundant, its energy equation is merely a linear combination of already listed 4 equations* (1.1-1.4) in the MS, for 3 ribbons and 1 triangle. This makes for indeterminacy 1 (degree of freedom α) and thus requires 1 closure equation.

In the lowest symmetry C_1 case, as *y*-crystal in Supplementary Figure 2b, the analysis is exactly the same except that for 8 unknowns one has 4 basic ribbons plus 2 triangles, that is 6 equations, with no more added by any larger polygons or shapes.

Supplementary Section-2c Adding cuts/edges expands the basic set, yet the same method works

The rank of MS increases but does not change the level of *indeterminacy* or degrees of freedom α's, which do not affect the shape, so the latter can still be predicted.

One substantial motivation to introduce extra cut(s) can be when empirical knowledge of a particular crystal suggests a specific facet, but which is not among the *a priori* basic edges.

Let us consider a general no-symmetry (C_1) case. A fragment of a polygon from Supplementary Figure 2b (Fig. 1a in the main text) is shown below in Supplementary Figure 3. Including a new cut (edges marked red n and blue n`) adds 2 new unknown variables $\varepsilon_n$ and $\varepsilon_{n`}$. In the MS set, one new equation to add obviously follows from a ribbon:

$\varepsilon_n + \varepsilon_{n`} = E_{nn`}$.

One more equation arises from a triangle formed by this cut and two edges from the prior set, for instance

$\varepsilon_1 + \varepsilon_3 + \varepsilon_{n`} = E_{13n`}$,

as shown, shaded pink. Any other extra-triangles would not add new nontrivial equations. A geometrical proof, although a bit tedious, is: such extra-triangle also containing n (or n') edge can be presented as a tiling combination (+/-) of the first-chosen triangle 13n` and a few from the prior set. That is by including an additional cut we add 2 unknown edge-energies, but also gain exactly 2 more equations in the MS, still in need of 2 closure equations containing auxiliary α and α` -- same 2 degrees of freedom.

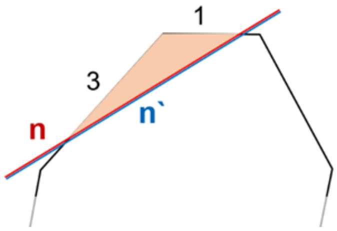

Supplementary Figure 3: A fragment of a polygon from Supplementary Figure 2b (Fig. 1a in the main text) including new cut, n`.

Another proof is as follows. Let us assume that for M cuts, that is a polygon with 2M sides-edges, the MS set contains M equations from the ribbons for each cut-direction, and M-2 equations from triangles, so 2 closure-equations make it fully determined (M + M-2 + 2 = 2M equations for 2M unknowns); for a selected fixed α, α`values one has exactly 2M equations and the linear system is fully determined. Now, adding one new "suggested" cut brings 2 variables (its edge energies) and also 2 equations (one ribbon, one triangle). Any other extra-triangle cannot add a nontrivial equation since this would make our system overdetermined, which is unphysical. So, we again conclude that even if the basic edges set is made larger to M → M+1, it merely expands the MS rank while preserving the number of closure equations 2 (or 1, for the C_2 symmetry case). Since we know it is true for small M =1, 2, 3, by mathematical induction it is true for any M, Q.E.D.

An important corollary: extra edges can be added to the polygonal construction, for approximating the edge-energy function $\varepsilon(a)$ to any desired accuracy (that is discretizing $\varepsilon(a)$ curve, in polar coordinates). This merely makes the algebraic MS larger, yet with the same couple of closure equations, so that solving such a linear system presents no principal difficulty, although is much more taxing and less elegant than using the IA. Importantly is shows that our interpolation anzats IA is not essential for solving the low-symmetry problem; it is definitely a convenient shortcut in going from small number of basis edges (8 in our approach to no-symmetry case C_1) to continuum representation of edge energy for arbitrary directions $\varepsilon(a)$, which is customary to use in discussion of Wulff construction. Moreover, an anzats (attempt, "trial answer") is often not expected to have a rigorous proof but is validated by practical use; in our case, all 2D materials considered in literature (graphene, hBN, several TMD, as cited in main text) and in this work produce rather satisfactory agreement with other theoretical predictions (done for higher symmetry cases) of with experimental images shown here.

Supplementary Section-3. Symbol correspondence between schematic and real crystals.

3.1 Symbols correspondence between Y-crystal edges and SnSe are

1 = *Z*Se, 1' = *Z*Sn, 2 = 2' = *A*, 3 = *A*Sn and 3' = *A*Se.

3.2 Symbols correspondence between *y*-crystal edges and $AgNO_2$ edge-angles are
1 = 0°, 1' = 180°, 2 = 79.5°, 2' = 259.5°, 3 = 48.5°, 3' = 228.5°, 4 = 117.2° and 4'= 297.2°

Supplementary Section-4. Lattice constants of the crystals.

4.1 For Y-crystal, the lattice parameters can be arbitrary but are chosen to match SnSe;
l_1 = 4.22 Å, l_2 = 4.52 Å, and l_3 = 6.19 Å (diagonal length)
$\angle\theta$ = 90° (angle between l_1 and l_2)

4.2 For SnSe, the lattice parameters are;
l_1 = 4.22 Å, l_2 = 4.52 Å, and l_3 = 6.19 Å (diagonal length)
$\angle\theta$ = 90° (angle between l_1 and l_2)

4.3 For SnS, the lattice parameters are;
l_1 = 4.02 Å, l_2 = 4.44 Å, and l_3 = 5.94 Å (diagonal length)
$\angle\theta$ = 90° (angle between l_1 and l_2)

4.4 For *y*-crystal, the lattice parameters can be arbitrary but are chosen to match $AgNO_2$;
l_1 = 3.39 Å and l_2 = 4.93 Å, and l_3 = 6.47 Å (along l_1+l_2), l_4 = 5.46 Å (along l_1-l_2)
$\angle\theta$ = 79.5° (angle between l_1 and l_2)

4.5 For $AgNO_2$, the lattice parameters are;
l_1 = 3.39 Å and l_2 = 4.93 Å, and l_3 = 6.47 Å (along l_1+l_2), l_4 = 5.46 Å (along l_1-l_2)
$\angle\theta$ = 79.5° (angle between l_1 and l_2)

Supplementary Section-5. Edge energy ε(*a*) interpolation ansatz (IA)

Wulff construction requires ε(*a*) for arbitrary direction (angle *a*) and for this we invoke an interpolation ansatz (IA): any slanted, vicinal edge is viewed as a sequence of small segments of the basic edges and, accordingly, its energy is decomposed into a sum of the basic energies, in proper proportions[25-27], like $c_1\varepsilon_1 + c_3\varepsilon_3$, etc. After straightforward trigonometry transformations, one can express the general ε(*a*) through basic EEs only and the geometry, i.e. the Bravais lattice parameters and angles[25]:

$$\varepsilon(a) = \varepsilon\cdot|\cos(a+C)|$$
$$\varepsilon = (c_1\varepsilon_i^2 + c_2\varepsilon_i\varepsilon_j + c_1\varepsilon_j^2)^{1/2}$$
$$C = \arctan[(c_3 + c_4\varepsilon_j/\varepsilon_{i.})/(c_5 + c_6\varepsilon_j/\varepsilon_i)]$$

Note that at basic edges directions it is exact, while at mid-angles the precision of this interpolation is not essential, usually having no effect on the resultant Wulff shape mostly defined by the minima of this function, which falls on the basic edges. The values of c_1 to c_6 parameters for the SnSe (and SnS) and $AgNO_2$ are given in Supplementary Tables 1 and 2 respectively.

IA holds as long as the basic edges chosen are lowest in energy. In case if the energy of any other edge due to specific reconstruction or passivation is found to be low, in computation or empirically observed, close or below the reconstructed basic edges and IA, such "new" edge should be added as the basic edge for IA, see also Supplementary Section-2c and the corollary. As we noted in the main text, this ever-possible issue is not specific to low-symmetry indeterminacy that we focus on, but is universal also for any high symmetry exploration. Further, as mentioned above in Supplementary Section-2c, for all 2D materials analyzed in literature (graphene, hBN, several TMD, cited in main text) and in this work, IA produces shapes in rather satisfactory agreement with other theoretical predictions (possible for higher symmetry cases, see hBN in Supplementary Section-10) or with experimental images, shown here.

Supplementary Table 1. The parameters of $\varepsilon(a)$ expression for Y-crystal (and SnSe, SnS).

a	ε_i	ε_j	c_1	c_2	c_3	c_4	c_5	c_6
$0° \leq a < 46°$	ε_1 (ε_{ZS})	ε_3 (ε_{ASn})	1.92	-2.66	0.96	-1.39	1	0
$46° \leq a < 90°$	ε_3 (ε_{ASn})	ε_2 (ε_A)	2.09	-3.01	0	1	-1.44	1.04
$90° \leq a < 134°$	$\varepsilon_{3'}$ (ε_{AS})	$\varepsilon_{3'}$ (ε_{AS})	2.09	-3.01	1	0	-1.04	1.44
$134° \leq a < 180°$	$\varepsilon_{3'}$ (ε_{AS})	ε_1 (ε_{ZSn})	1.92	-2.66	1.39	-0.96	0	1

Supplementary Table 2. The parameters of $\varepsilon(a)$ expression for *y*-crystal (and $AgNO_2$).

a	ε_i	ε_j	c_1	c_2	c_3	c_4	c_5	c_6
$0° \leq a < 48.5°$	ε_3	ε_1	1.78	-2.36	-1.335	0.885	0	1
$48.5° \leq a < 79.5°$	ε_2	ε_3	3.77	-6.46	-1.287	0.354	-1.454	1.909
$79.5° \leq a < 117.2°$	ε_4	ε_2	2.67	-4.23	0.298	0.748	1.609	-1.454
$117.2° \leq a < 180°$	ε_4	$\varepsilon_{1'}$	1.26	-1.16	1.125	-0.514	0	1
$180° \leq a < 228.5°$	$\varepsilon_{3'}$	$\varepsilon_{1'}$	1.78	-2.36	-1.335	0.885	0	1
$228.5° \leq a < 259.5°$	$\varepsilon_{2'}$	$\varepsilon_{3'}$	3.77	-6.46	-1.287	0.354	-1.454	1.909
$259.5° \leq a < 297°$	$\varepsilon_{4'}$	$\varepsilon_{2'}$	2.67	-4.23	0.298	0.748	1.609	-1.454
$297° \leq a < 360°$	$\varepsilon_{4'}$	ε_1	1.26	-1.16	1.125	-0.514	0	1

Supplementary Section-6. Master system (MS), its right-hand side (RHS) parameters, and example edge structures.

We estimate the RHS of the master system of equations like (1.1-1.4) by DFT total energy calculations of the 2D shapes, with edges, taken *relative* to that of the corresponding bulk crystal phase, or the exact same numbers N_a, N_b, … of each constituent element-atom, with their appropriate thermodynamic chemical potentials μ_a, μ_b, ... Note that, such an atom-precise subtraction corresponds to a proper Gibbs surface choice in the treatment of continuum-gradual interfaces[38, 39]. Subtracting exact same number of atoms, i.e. moles, of each element is also fully in accord with the *equimolar cut* requirement[5,40] in the atomistic models comparing a cluster with a geometrical volume cut out of a bulk phase, to determine energy excess contribution from the surface (perimeter, in case of 2D). Consider a bi-elemental 2D layer with elements 'a' and 'b', of 1:1 stoichiometry. The energy of the perimeter of any shape of this 2D layer can be expressed as: $E_{perimeter} = E_{total}(2D\ shape) - \mu_a N_a - \mu_b N_b$, where μ_a and μ_b are the chemical potentials of 'a' and 'b'. Note

that the sum, $\mu_{ab} = \mu_a + \mu_b$ is fixed to the energy of the 'ab'-phase present (3D or 2D form), which is directly computable with standard DFT (or empirical-atomistic potentials) as $\mu_{ab} = E_{bulk}$/cell. Remaining degree of freedom is either one of the elemental chemical potentials, or for the convenience of keeping things symmetric, their difference, $\mu = ½(\mu_a - \mu_b)$. Dictated by equilibrium with the environment, a certain assumed value of μ can change the RHS and accordingly affect the crystal shape, as we illustrate in case of SnS and SnSe, below and in the main text, Fig. 3. For practical use, it is convenient to rewrite the RHS in the MS, that is the perimeter energy, as $E_{perimeter} = E_{total}(\text{shape}) - \mu_a N_a - \mu_b N_b = E_{total}(\text{shape}) - ½\mu_{ab}(N_a+N_b) - \mu(N_a-N_b)$. In the latter, the first two terms are exactly computable, while the last one reflects the elemental imbalance from exact stoichiometry ($N_a=N_b$) and depends on the environment's chemical potential. All the energies on the RHS are estimated using DFT of the appropriate geometries (i.e. triangles and ribbons) whose perimeter is composed of the edges, while μ can be chosen according to the conditions (e.g. high for Se-rich or low for Sn-rich conditions, in case of SnSe crystal).

For example,

1) In the case of ribbons with edges 1 and 1', the master equation is estimated as follows.

$l_1\varepsilon_1 + l_{1'}\,\varepsilon_{1'} = E_{total}(\text{ribbon}) - \mu_a N_a - \mu_b N_b$, where $l_1 = l_{1'}$ are the lengths of the edges.

2) In the case of a triangle with edges 1, 2 and 3, with lengths l_1, l_2 and l_3 the master equation is as below.

$l_1\varepsilon_1 + l_2\,\varepsilon_2 + l_3\,\varepsilon_3 = E_{total}(\triangle) - \mu_a N_a - \mu_b N_b$

In addition to calculating the energies of ribbons with different edge terminations, we have also considered edge-reconstruction depending on the chemical potential (μ). In doing so, we have constructed several different structures for a given edge. E.g. for Sn rich conditions, the edge has Se vacancies (in varying concentrations), and for Se rich conditions we simulate excess Se-adatoms (in varying concentrations). Then we utilize only those reconstructed edges that yielded the lowest energies at a given μ.

6.1 For Y-crystal, the right hand side values can be chosen arbitrarily, as examples, but we select the values to be similar to SnSe (computed using DFT), so the master system is, for the ribbons:

$\varepsilon_1 + \varepsilon_{1'} = 0.136$ eV/Å or $(\varepsilon_1 + \varepsilon_{1'})l_1 = 0.574$ eV (S1)

$\varepsilon_2 = 0.103$ eV/Å or $\varepsilon_2\, l_2 = 0.466$ eV

$\varepsilon_3 + \varepsilon_{3'} = 0.097$ eV/Å or $(\varepsilon_3 + \varepsilon_{3'})l_3 = 0.600$ eV

and for the triangle:

$4.22\varepsilon_1 + 4.52\varepsilon_2 + 6.19\varepsilon_{3'} = 1.11$ eV or $\varepsilon_1 + 1.07\varepsilon_2 + 1.47\varepsilon_{3'} = 0.263$ eV

6.2 For SnSe, the master system's RHS values are computed with DFT.

6.2.1 Element-balanced edges (corresponding to zero slope, independent of μ, in main text Fig. 3b). Its atomistic structure is shown in figure below (orange balls represent Se atoms and gray for Sn), as example, and accordingly the MS equations are:

$\varepsilon_1 + \varepsilon_{1'} = 0.164$ eV/Å

$\varepsilon_2 = 0.103$ eV/Å

$\varepsilon_3 + \varepsilon_{3'} = 0.097$ eV/Å

$\varepsilon_1 + 1.07\varepsilon_2 + 1.47\varepsilon_{3'} = 0.263$ eV

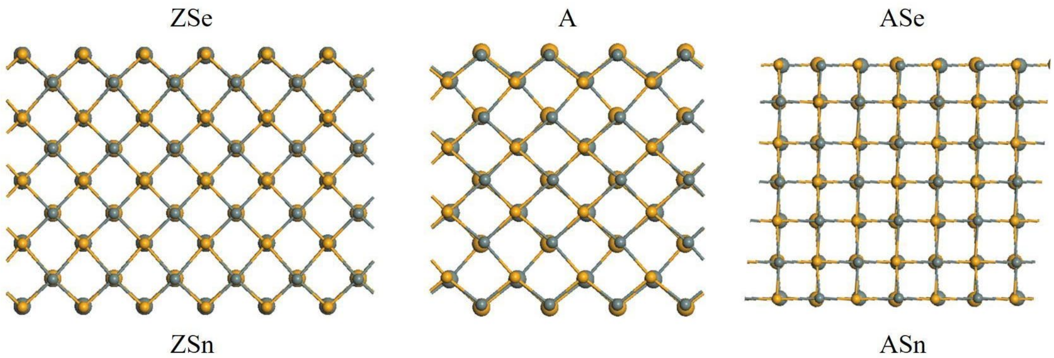

Supplementary Figure 4: Element-balanced edges for SnSe. orange balls are Se and gray are Sn atoms.

6.2.2 Sn-rich edges (corresponding to positive, upward slope in Fig. 3b)

$\varepsilon_1 + \varepsilon_{1'} = 0.474\mu_{Se} + 2.367$ eV/Å (S2)

$\varepsilon_2 = 0.103$ eV/Å

$\varepsilon_3 + \varepsilon_{3'} = 0.097$ eV/Å

$\varepsilon_1 + 1.07\varepsilon_2 + 1.47\varepsilon_{3'} = 0.263$ eV

6.2.3 Se-light-rich edges (corresponding to smaller negative, downward slope in Fig. 3b)

$\varepsilon_1 + \varepsilon_{1'} = 0.164$ eV/Å

$\varepsilon_2 = - 0.221\mu_{Se} - 0.674$ eV/Å

$\varepsilon_3 + \varepsilon_{3'} = 0.097$ eV/Å

$\varepsilon_1 + 1.07\varepsilon_2 + 1.47\varepsilon_{3'} = - 0.237\mu_{Se} - 0.570$ eV

6.2.4 Se-heavy-rich edges (corresponding to larger negative, downward slope in Fig. 3b)

$\varepsilon_1 + \varepsilon_{1'} = - 0.711\mu_{Se} - 2.239$ eV/Å

$\varepsilon_2 = - 0.221\mu_{Se} - 0.674$ eV/Å

$\varepsilon_3 + \varepsilon_{3'} = 0.097$ eV/Å

$\varepsilon_1 + 1.07\varepsilon_2 + 1.47\varepsilon_{3'} = - 0.711\mu_{Se} - 2.172$ eV

6.3 For SnS, the master system's RHS values are computed with DFT.

6.3.1 Element-balanced edges (corresponding to zero slope, independent of μ, in Supplementary Figure 6a). Its atomistic structure is shown in figure below (yellow balls represent S atoms and grey for Sn), as example, and accordingly the MS equations are:

$\varepsilon_1 + \varepsilon_{1'} = 0.176$ eV/Å

$\varepsilon_2 = 0.119$ eV/Å

$\varepsilon_3 + \varepsilon_{3'} = 0.100$ eV/Å

$\varepsilon_1 + 1.10\varepsilon_2 + 1.48\varepsilon_{3'} = 0.293$ eV

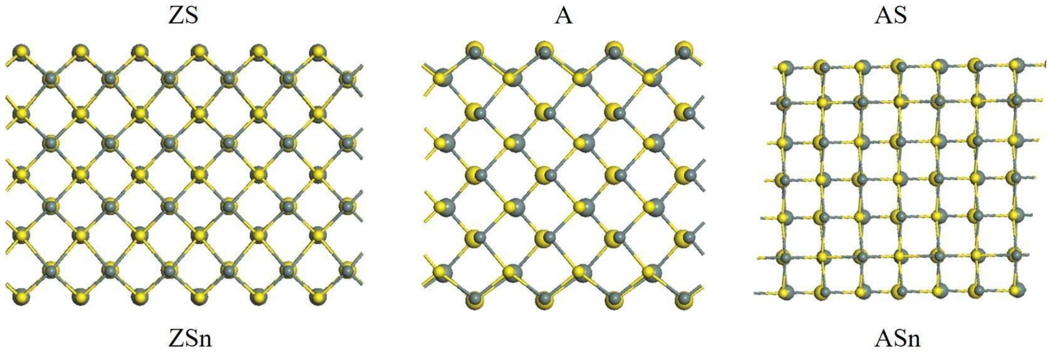

Supplementary Figure 5: Element-balanced edges for SnS. yellow balls are for S and grey for Sn atoms.

6.3.2 Sn-light-rich edges (corresponding to smaller positive, upward slope in Supplementary Figure 9a)
$\varepsilon_1 + \varepsilon_{1'} = 0.176$ eV/Å
$\varepsilon_2 = 0.375\mu_S + 2.177$ eV/Å
$\varepsilon_3 + \varepsilon_{3'} = 0.100$ eV/Å
$\varepsilon_1 + 1.10\varepsilon_2 + 1.48\varepsilon_{3'} = 0.395\mu_S + 2.459$ eV
6.3.3 Sn-heavy-rich edges (corresponding to larger positive, upward slope in Supplementary Figure 9a)
$\varepsilon_1 + \varepsilon_{1'} = 0.663\mu_S + 3.854$ eV/Å
$\varepsilon_2 = 0.375\mu_S + 2.177$ eV/Å
$\varepsilon_3 + \varepsilon_{3'} = 0.100$ eV/Å
$\varepsilon_1 + 1.10\varepsilon_2 + 1.48\varepsilon_{3'} = 0.711\mu_S + 4.212$ eV
6.3.4 S-light-rich edges (correspond to smaller negative, downward slope in Supplementary Figure 9a)
$\varepsilon_1 + \varepsilon_{1'} = 0.176$ eV/Å
$\varepsilon_2 = -0.225\mu_S - 0.813$ eV/Å
$\varepsilon_3 + \varepsilon_{3'} = 0.100$ eV/Å
$\varepsilon_1 + 1.10\varepsilon_2 + 1.48\varepsilon_{3'} = -0.249\mu_S - 0.738$ eV
6.3.5 S-heavy-rich edges (corresponding to larger negative, downward slope in Supplementary Figure 9a)
$\varepsilon_1 + \varepsilon_{1'} = -0.746\mu_S - 2.790$ eV/Å
$\varepsilon_2 = -0.225\mu_S - 0.813$ eV/Å
$\varepsilon_3 + \varepsilon_{3'} = 0.100$ eV/Å
$\varepsilon_1 + 1.10\varepsilon_2 + 1.48\varepsilon_{3'} = -0.746\mu_S - 2.714$ eV
6.4 For *y*-crystal, chemically unspecified, so the right hand side RHS values for the MS equations have been chosen arbitrarily. The master system is:
<u>ribbons</u>
$\varepsilon_1 + \varepsilon_{1'} = 0.5$ eV/Å (S3)
$\varepsilon_2 + \varepsilon_{2'} = 0.7$ eV/Å
$\varepsilon_3 + \varepsilon_{3'} = 0.6$ eV/Å
$\varepsilon_4 + \varepsilon_{4'} = 0.8$ eV/Å
triangles
$3.39\varepsilon_1 + 4.93\,\varepsilon_2 + 6.47\,\varepsilon_{3'} = 5.1$ eV
$3.39\,\varepsilon_1 + 5.46\,\varepsilon_4 + 4.93\,\varepsilon_{2'} = 5.4$ eV
6.5 For $AgNO_2$, chemically-specific material, the master system's RHS values are computed with DFT.
Definition of μ,
$\mu_{Ag} + \mu_{NO2} = \mu_{AgNO2}$
Fix $\mu_{Ag} = \mu(\text{Ag bulk}) = -2.81$ eV
ribbons
$l_1\,(\varepsilon_1 + \varepsilon_{1'}) = E(\text{ribbon})_1 - 2\,\mu_{AgNO2} - \mu_{Ag}$
$l_2\,(\varepsilon_2 + \varepsilon_{2'}) = E(\text{ribbon})_2 - 4\,\mu_{AgNO2}$
$l_3\,(\varepsilon_3 + \varepsilon_{3'}) = E(\text{ribbon})_3 - 5\,\mu_{AgNO2} - \mu_{Ag}$
$l_4\,(\varepsilon_4 + \varepsilon_{4'}) = E(\text{ribbon})_4 - 4\,\mu_{AgNO2} - \mu_{Ag}$
triangles
$(6 - 5) \times (l_1\,\varepsilon_1 + l_2\,\varepsilon_{2'} + l_4\,\varepsilon_4) = E(6\triangle)_a - E(5\triangle)_a - 7\,\mu_{AgNO2} - \mu_{Ag}$
$(6 - 5) \times (l_1\,\varepsilon_1 + l_2\,\varepsilon_2 + l_3\,\varepsilon_{3'}) = E(6\triangle)_b - E(5\triangle)_b - 7\,\mu_{AgNO2} - \mu_{Ag}$

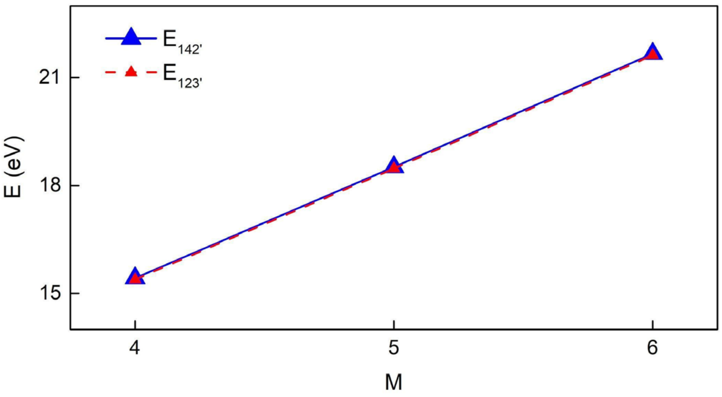

Supplementary Figure 6. Variation of [E(M△) – N μ_{AgNO2} – N' μ_{Ag}] in $AgNO_2$, for both a (142') and b(123') triangles for μ_{Ag}= –2.81 eV. The energy scales linearly with size M, as E ~ 2.9 + 3.2*M.

Below is the final MS after substitution of DFT energies for ribbons and triangles at μ_{Ag} = –2.81 eV.

ribbons

$\varepsilon_1 + \varepsilon_{1'} = 0.82$ eV/Å (S4)

$\varepsilon_2 + \varepsilon_{2'} = 0.01$ eV/Å

$\varepsilon_3 + \varepsilon_{3'} = 0.52$ eV/Å

$\varepsilon_4 + \varepsilon_{4'} = 0.64$ eV/Å

triangles

$3.39\varepsilon_1 + 4.93\ \varepsilon_2 + 6.47\ \varepsilon_{3'} = 3.15$ eV

$3.39\ \varepsilon_1 + 5.46\ \varepsilon_4 + 4.93\ \varepsilon_{2'} = 3.15$ eV

Supplementary Section-7. Details to accompany Fig. 3 in the main text.

Experimental images of SnSe, insets above Fig. 3b: the yellow rhomb image is adapted from[29] and the violet rectangle and also violet two-corner truncated rectangle are both from[30].

Since the rhomb-shape (yellow, above Fig. 3c) remains invariant within a relatively wide range (-0.6, 0.7) of chemical potential; we omitted most of that interval, as noted by the broken curves and the axes in Fig. 3c. Also, the expression used to illustrate the α(μ) arbitrariness in the segment μ < -0.59 was altered in sign, to reduce disruption in curves. To provide a broader behavior we include here an uninterrupted plot.

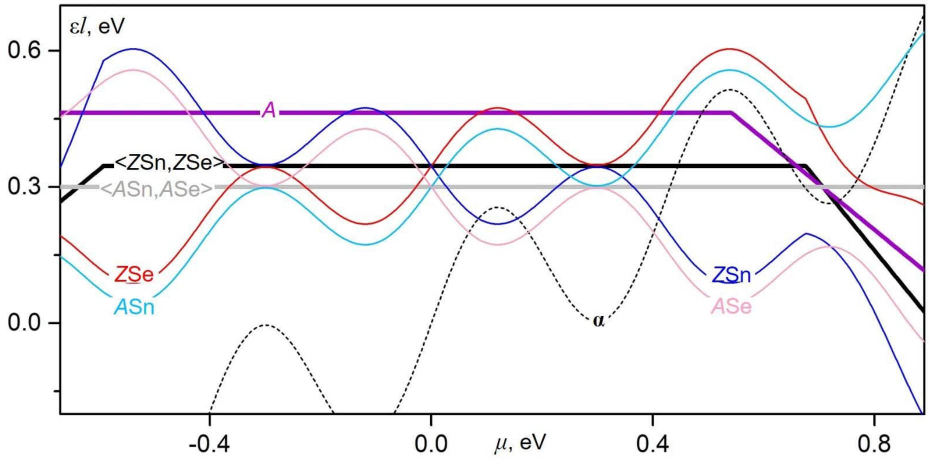

Supplementary Figure 7. The auxiliary EE, with a random choice of $\alpha l = 0.62\mu + 0.19 \sin 15\mu$, shown for the continuous μ range (-0.7, 0.9).

Supplementary Section-8. SnS Wulff shapes.
Here we show that the effect of edge reconstruction and passivation can be included in the analysis, without any change to the main idea of our method. Including such refinements allows one to identify edge structures that are more realistic for specific materials and conditions, if so desired. Performing more careful edge-reconstruction, and within the broader chemical potential range, reveals/predicts additional shapes, which has also been observed in experiment. This does not constitute the "proof of the method" (which is essentially ab initio) because the exact experimental conditions were not documented in the literature. Yet such agreement strongly corroborates the method validity and practicality.

Supplementary Figure 8 shows the variation in the shape of SnS without edge passivation, i.e. we neglect an excess of S or Sn at the edges. This results in formally/mathematically correct but unreasonably (from physics viewpoint) large EEs, seen as a long petal in Supplementary Figure 8a. Most importantly, the shapes predicted without the right passivation/reconstruction cannot reproduce some of the experimentally grown morphologies. On the other hand, when we do passivate the edges with Sn or S atoms, in accord to μ, we can resolve additional morphologies that agree with more experimental shapes (Supplementary Figure 9), with EEs notably smaller than in the unpassivated case. Since the exact edge reconstruction/passivation is not known, we make our best guess of it guided by reducing the energy and from literature[41] and find that it reproduces experimental shapes quite accurately.

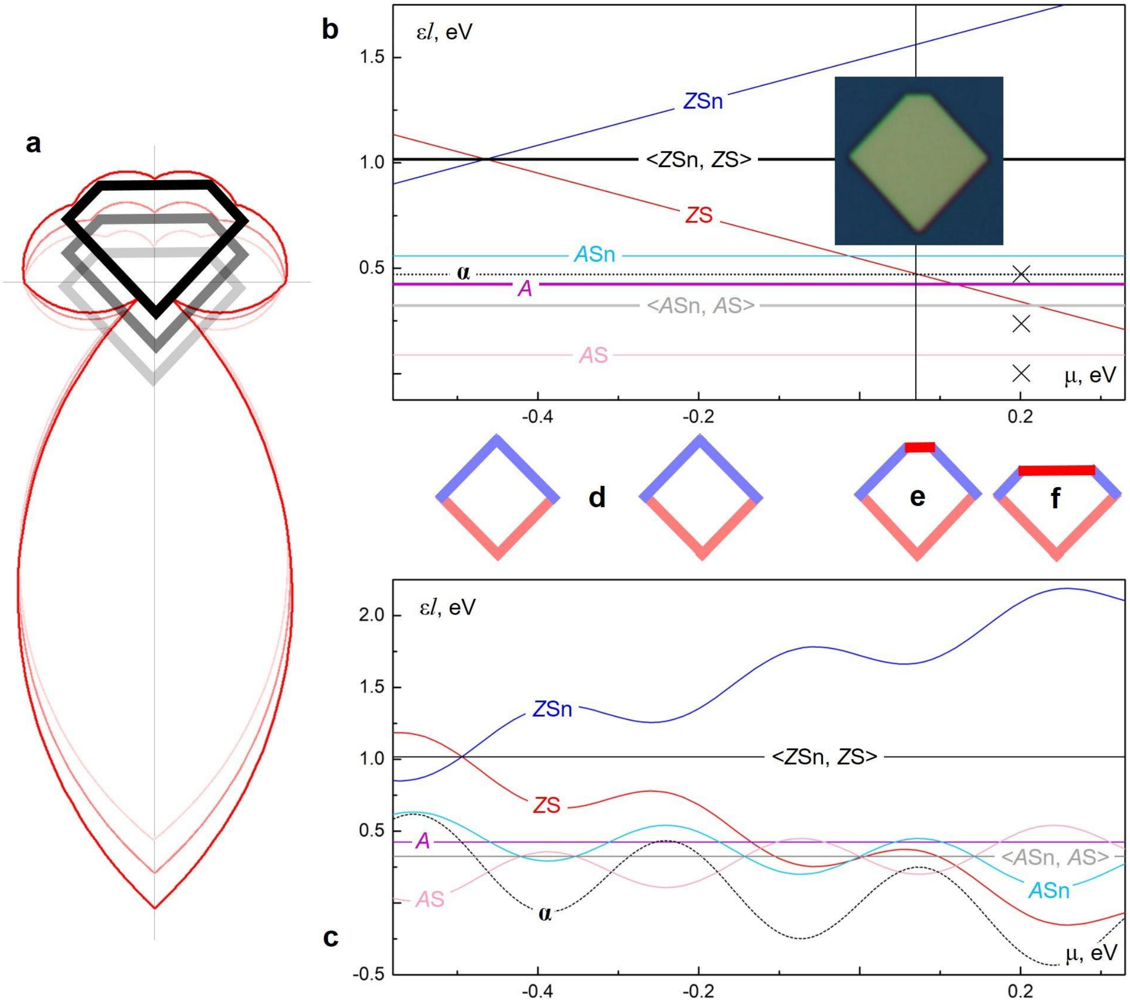

Supplementary Figure 8. The auxiliary edge energies and Wulff constructions of SnS without edge-reconstruction. (**a**) The ε-plots (red) and the Wulff shapes (black) of SnS, at $\lambda \equiv \varepsilon_3 - \varepsilon_{3'} = 0, 0.04, 0.08$. (**b**)

The auxiliary edge energies of SnS varying with chemical potential, at $\lambda \equiv \varepsilon_3 - \varepsilon_{3'} = 0.08$. Black crosses correspond to shapes in (a) or (f), while the vertical line corresponds to **e** or the experimental image[42] in the inset of (b). (**c**) The auxiliary EE chosen at random as $\alpha l = -0.59\mu + 0.29 \sin 20\mu$. For the SnS Wulff constructions, $\mu = -0.4$ and -0.2 in (**d**), $\mu = 0.07$ in (**e**), and $\mu = 0.2$ in (**f**); Thick red, light-red and light-blue lines represent *Z*S, *A*S and *A*Sn edges, respectively.

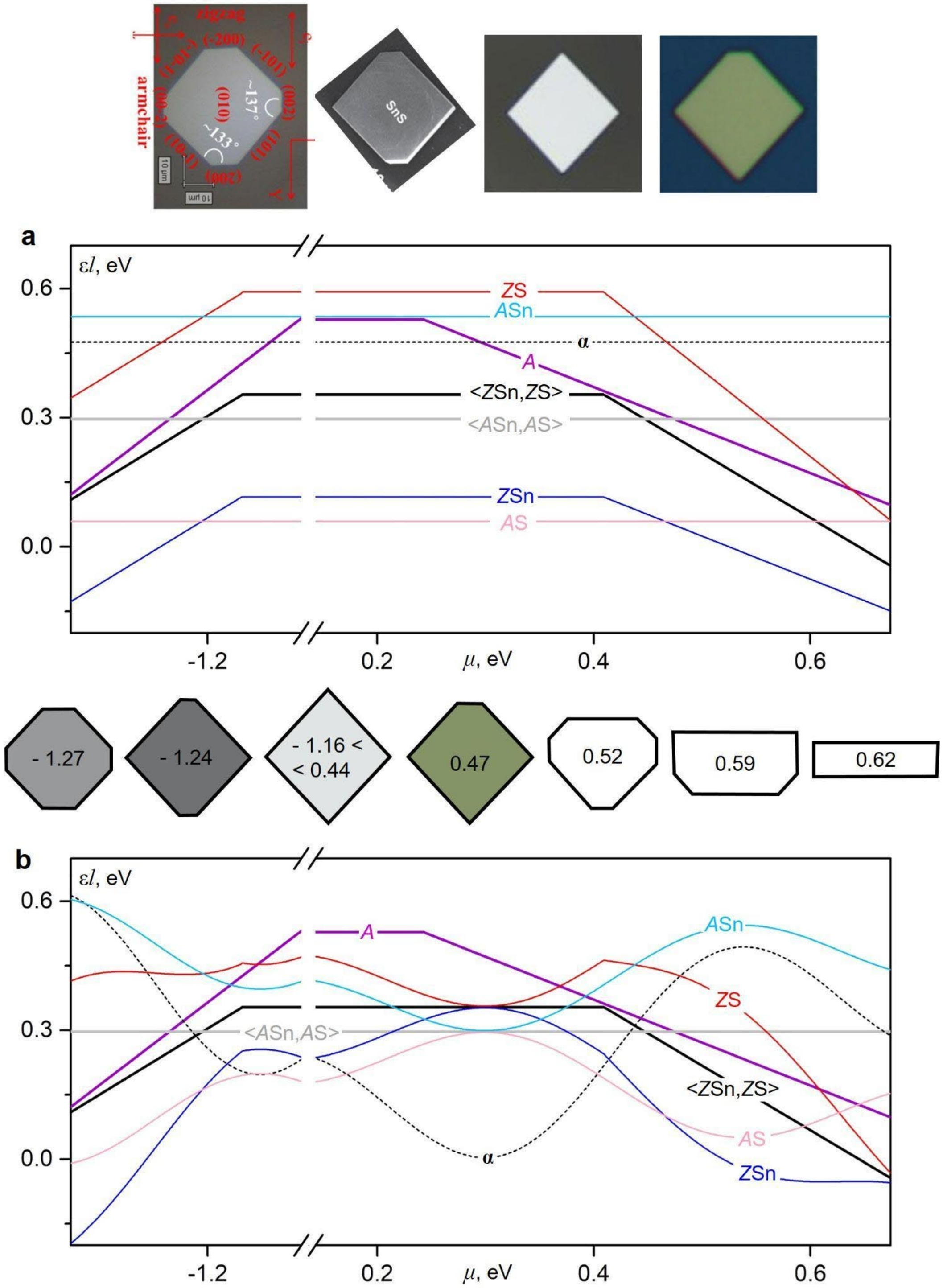

Supplementary Figure 9. The auxiliary edge energies and Wulff constructions of SnS. (**a**) The auxiliary edge energies of SnS vary with chemical potential, at $\alpha \equiv \varepsilon_3 - \varepsilon_{3'} = 0.08$. Numbers on bottom shapes are

values of μ. Above inserted are the experimental images adapted from[42-44]. (**b**) The auxiliary EE chosen at random as $\alpha l = 0.594\mu + 0.178 \sin 15\mu$, for $\mu \geq -1.1$ while $\alpha l = -0.327\mu - 0.178 \sin 15\mu$, to reduce disruption in curves at $\mu < -1.1$ (note that changing α does not affect the identified shapes).

From Fig. 3 b,c (main text) and Supplementary Figure 9 a,b, we can see that both SnSe and SnS have element-balanced edges as the most stable ones in a wide chemical potential μ range (it's -0.6 to 0.7 eV for SnSe, and -1.16 to 0.44 eV for SnS), where the equilibrium shape is a rhombus. It means that the shape most stable for a wide range of the chemical potential, and hence most probable for these two materials is a rhombus. The element-balanced edges result in constant energies of the ribbons along basic edge directions (Z, A, and A'). While under extreme chemical potential conditions (Sn-rich or Se-/S- rich), the element-unbalanced edges reach lower energy in both materials, which introduces chemical potential dependent and competing Z/A/A' edges with lower energies, and hence the rhombus shape is lost. Due to different edge reconstructions, SnSe has a rectangle, a two-corners-truncated rectangle, a non-regular octagon, and a one-corner-truncated rhombus as equilibrium shapes under extreme chemical potential conditions, shown in Fig. 3; while SnS has 2 more shapes than SnSe, a two-corners-truncated rhombus and an octagon, shown in Supplementary Figure 9.

Supplementary Section-9. Symmetry classification.

Apart from the more popular 2D materials with well defined edge energies, such as graphene, hBN or metal dichalcogenides, there exist several less studied 2D van der Waals materials that lack the sufficient symmetry to define their edge energies, making their shapes theoretically unpredictable. Some such materials are SnS, SnSe, $GeAs_2$, SiP_2, AsO_2, SnTe, GaTeCl, $VOBr_2$, $VOCl_2$, As_2O_3, I_2O_5, In_2Se_3, $LiBH_4$, YbP_5 and $AgNO_2$, all of them predicted to be exfoliable into 2D layers. In particular, SnSe which exists as 2D flakes[39] is especially important, for its ability to exhibit exceptional thermoelectric properties with very high ZT merit in bulk[19-24]. Hence, formulating a strategy to predict the shapes of these important materials is not only of interest for fundamental physics, but also for predicting the change in structural and electronic properties of the material that accompany the change in shape.

Here we show that all 2D materials can be grouped into 4 types according to the determinacy of edge energy as Supplementary Table 3, and classify 230 space groups into the 4 types as Supplementary Table 4.

Supplementary Table 3. The types of 2D materials according to the determinacy of ε.

Types	The determinacy of ε	Inversion symmetry	The number of directions of solvable ε
W_i	well defined	Yes	>2
W_n	well defined	No	>2
N_2	not well defined	No	2
N_0	not well defined	No	0

Supplementary Table 4. The classification of 230 space groups according to the determinacy of ε (2D materials types in Supplementary Table 3). Here x, y, z are directions normal to the cuts of the 3D bulk yielding the 2D material-layers. Hence, the normal to the 2D plane will be along either of these directions, and the 3D space groups belonging to the point groups are given in the brackets.

	x	y	z
W_i	C_i(2), C_{2h}(10-15), D_{2h}(47-74), C_{4h}(83-88), D_{4h}(123-142), S_6(147, 148), D_{3d}(162-167), D_{6h}(191-194), T_h(200-206), O_h(221-230)	C_i(2), C_{2h}(10-15), D_{2h}(47-74), C_{4h}(83-88), D_{4h}(123-142), S_6(147, 148), D_{3d}(162-167), D_{6h}(191-194), T_h(200-206), O_h(221-230)	C_i(2), C_{2h}(10-15), D_{2h}(47-74), C_{4h}(83-88), D_{4h}(123-142), S_6(147, 148), D_{3d}(162-167), D_{6h}(191-194), T_h(200-206), O_h(221-230)
W_n	D_2(16-24), D_4(89-98), D_{2d}(111-116, 119-122), D_3(150, 152, 154, 155), D_{3h}(187-190), T(195-199), O(207-214), T_d(215-220)	C_2(3, 5), D_2(16-24), D_4(89-98), D_{2d}(111-116, 119-122), D_3(150, 152, 154, 155), D_{3h}(187-190), T(195-199), O(207-214), T_d(215-220)	D_2(16-24), C_{2v}(25-46), C_4(75, 77, 79), S_4(81, 82), D_4(89-98), C_{4v}(99-110), D_{2d}(111-122), C_3(143, 146), D_3(149-155), C_{3v}(156-161), C_6(168, 171-173), C_{3h}(174), C_{6v}(183-186), D_{3h}(187-190), T(195-199), O(207-214), T_d(215-220)

N_2	C_2(3-5), C_s(6-9), C_{2v}(25-46), C_4(75-80), S_4(81, 82), C_{4v}(99-110), D_{2d}(117, 118), D_3(149, 151, 1535), C_{3v}(156, 158, 160, 161), C_6(168-173), C_{3h}(174), C_{6v}(183-186)	C_{2v}(25-46), C_4(75-80), S_4(81, 82), C_{4v}(99-110), D_{2d}(117, 118), D_3(149, 151, 153), C_{3v}(156, 158, 160, 161), C_6(168-173), C_{3h}(174), C_{6v}(183-186)	C_2(3-5), C_s(6-9)
N_0	C_1(1), C_3(143-146), C_{3v}(157, 159)	C_1(1), C_2(4), C_s(6-9), C_3(143-146), C_{3v}(157, 159)	C_1(1), C_4(76, 78, 80), C_3(144, 145), C_6(169, 170)

Supplementary Section-10. hBN Wulff shapes.

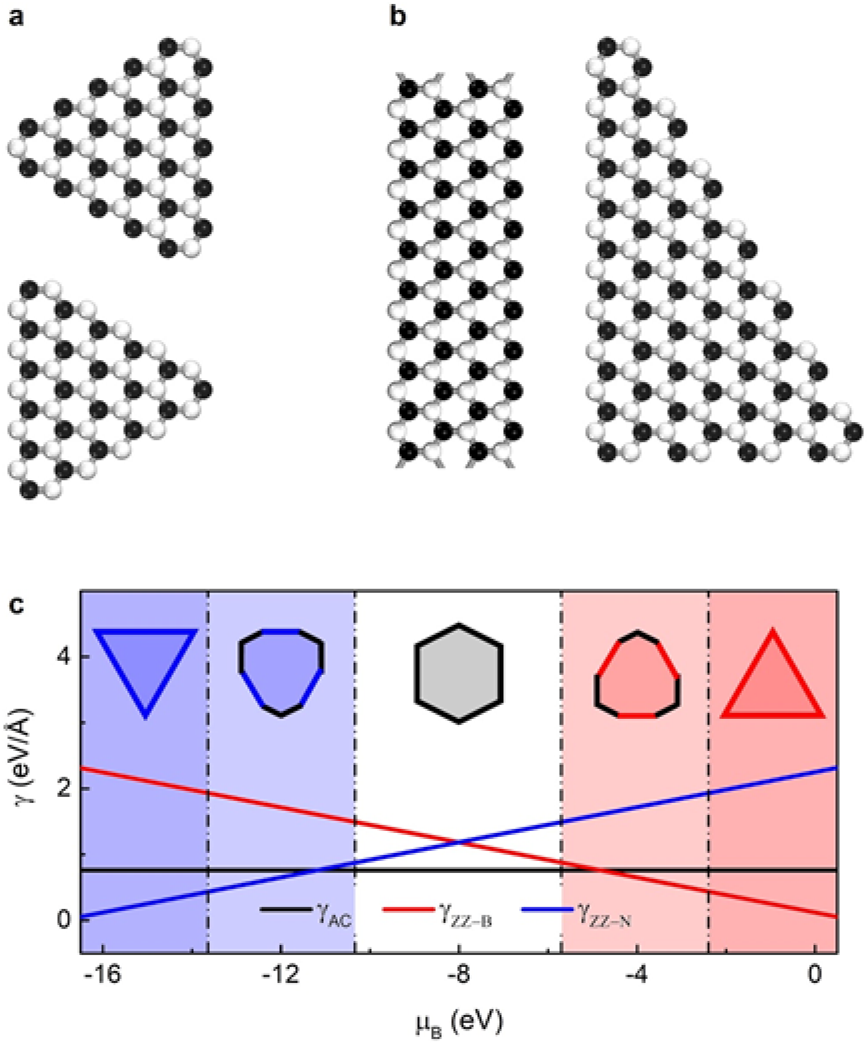

Supplementary Figure 10. Structure, edge energies and ES of BN. **(a)** BN triangles with identical edges used in previous work. **(b)** BN ribbon and triangle with different edges used in this work. **(c)** edge energies of AC, ZZ-B and ZZ-N edges as chemical potential of sulfur μ_B varies. Inserted polygons are ES corresponding to different μ_B ranges, which are separated by dot dash line and shaded in different colors, respectively.

As established from our analysis and examples, the "auxiliary edge energy" method can determine the ES of 2D low-symmetry materials with no-well defined edge energy; where some edges can never be isolated and hence their energies never resolved. We note that a situation when a particle is in contact with a substrate leads to additionally reduced symmetry, but the treatment has always been fully based on fully known surface and interface (contact) energies. This approach, often known as Winterbottom[45] or Wulff-Kaischew[46] construction, is just a minor modification of the Wulff theorem. It also extends the traditional Wulff construction use by overcoming the limitation that the ES can only be determined for materials with separable edges. To establish the validity and gain new perspective into the relationship between "auxiliary edge energy" method and traditional ES determining techniques, we apply it to sufficient symmetry materials for which the solution for ES is readily available[11]. Here, we apply our method to monolayer hexagonal boron nitride (BN).

It's well-known that BN has D_{3h} symmetry, and three basic directions, A, Z-B and Z-N. The edge energy $\varepsilon_{BN}(a)$ along an arbitrary orientation can be expressed as interpolation ansatz (IA) in Supplementary Section-5:

$\varepsilon(a) = |\varepsilon| \cos(a + C)$

where $|\varepsilon| = 2\,(\varepsilon_A^2 + \varepsilon_{Zx}^2 - \sqrt{3}\,\varepsilon_A\,\varepsilon_{Zx})^{1/2}$ and $C = \mathrm{sgn}(a) \cdot \arctan(\sqrt{3} - 2\,\varepsilon_{Zx}/\varepsilon_A)$ (S5)

with the subscript x = N or x = B. Since Z-B and Z-N edges can't be separated by a ribbon, they were in previous work calculated using an equilateral triangle with identical edges[11] (Supplementary Figure 10a). However, in the "auxiliary edge energy" method the determination of ε_{basic} skips the construction of polygons with identical edges, i.e. it overcomes the necessity of isolating all the edges. Instead, we use a ribbon with opposite Z-N and Z-B edges, as seen in Supplementary Figure 10b. On the other hand, the A edge can be isolated with a ribbon, and $\varepsilon_A = 0.76$ eV/Å is estimated, in agreement with previous work[11].

Although the A edge is independent of μ, the dependence of Z edges on μ affects the delicate balance between ε_{basic}, which changes the ES of BN. In order to establish the relation between $\varepsilon_{Z\text{-}B}$, $\varepsilon_{Z\text{-}N}$ and μ_B (chemical potential of B), we construct a triangle with *non-identical edges*, an advantage of the "auxiliary energy method", as shown in Supplementary Figure 10b. We arrive at the same expressions as Liu *et al.*[11], $\varepsilon_{Z\text{-}B} = (0.12 - 0.13\mu_B)$ eV/Å and $\varepsilon_{Z\text{-}N} = (0.12 + 0.13\mu_B)$ eV/Å. These $\varepsilon_{basic}(\mu_B)$ when substituted in the equation for ε, reproduce ES of BN as a function of μ_B as shown in Supplementary Figure 10c (in agreement with previous results[11]. We find that from low to high μ_B, ES evolves from triangle with Z-N edges → enneagon with Z-N and A edges → hexagon with A edges → enneagon with Z-B and A edges → triangle with Z-B edges. Thus, confirming the validity and applicability of our "auxiliary edge energy" method.

Supplementary Section-11. The 23 equations for 3D crystals.

With Supplementary Figure 11 below, axes are **1**, **2**, **3**, and notations of the opposites are **1'** for **-1**, **2'** for **-2**, and **3'**for **-3**. C_i are constants. One can refer to the surfaces by modifying the Miller indices (h,k,l), i.e. (1,0,0) is 1, (1,1,0) is 12, and (1,1,1) is 123, etc.

Therefore, there are 6 basic surfaces 1, 2, and 3 (and their opposites 1', 2', and 3').

There are 12 basic diagonal surfaces accordingly 12, 1’2 (and their opposites 1’2’, 12’), the 23, 2’3 (2’3’ and 23’), and 31, 3’1 (3’1’, 31’).
Finally, there are 8 body diagonal basic surfaces are 123, 1’2’3, 1’23, 12’3 (and their opposites, 1’2’3’, 123’, 12’3’, 1’23’).
This way, in 3D one has 26 (6+12+8) unknowns, the energies for all the basic facets.
23 surface energy (SE) equations (13 slabs, 6 wedges, 4 tetrahedrons).

13 slabs:

The first 3 slab energy equations are defined by the pairs of parallel basic surfaces given by 1/1’ or 2/2’ or 3/3’, where ‘/’ denotes pairing. The basic SE equations for this case are as follows.

$\varepsilon_1+\varepsilon_{1'}= C_1$, $\varepsilon_2+\varepsilon_{2'}=C_2$, $\varepsilon_3+\varepsilon_{3'}=C_3$,

Similarly, the remaining 10 slabs are defined by 6 pairs of parallel diagonal basic surfaces given by (12/1’2’ or 12’/1’2 etc) and 4 slabs of basic body diagonal surfaces given by (123/1’2’3’ or 123’/1’2’3 etc.) The basic SE equations for this case are as follows.

$\varepsilon_{12}+\varepsilon_{1'2'}=C_4$, $\varepsilon_{12'}+\varepsilon_{1'2}=C_5$,
$\varepsilon_{23}+\varepsilon_{2'3'}=C_6$, $\varepsilon_{23'}+\varepsilon_{2'3}=C_7$,
$\varepsilon_{31}+\varepsilon_{3'1'}=C_8$, $\varepsilon_{31'}+\varepsilon_{3'1}=C_9$,
$\varepsilon_{123}+\varepsilon_{1'2'3'}=C_{10}$, $\varepsilon_{123'}+\varepsilon_{1'2'3}=C_{11}$,
$\varepsilon_{12'3'}+\varepsilon_{1'23}=C_{12}$, $\varepsilon_{12'3}+\varepsilon_{1'23'}=C_{13}$,

6 wedges:

The wedges are volumes enclosed by two non-parallel basic surfaces and one diagonal basic surface given by (1/2/1’2’ etc). The basic SE equations for this case are as follows.

$\varepsilon_1+\varepsilon_2+\varepsilon_{1'2'}=C_{14}$, $\varepsilon_{1'}+\varepsilon_2+\varepsilon_{12'}=C_{15}$,
$\varepsilon_2+\varepsilon_3+\varepsilon_{2'3'}=C_{16}$, $\varepsilon_{2'}+\varepsilon_3+\varepsilon_{23'}=C_{17}$,
$\varepsilon_3+\varepsilon_1+\varepsilon_{3'1'}=C_{18}$, $\varepsilon_{3'}+\varepsilon_1+\varepsilon_{31'}=C_{19}$,

4 tetrahedrons:

The tetrahedrons are formed at the corners of each quadrant; enclosed by 3 basic surfaces and one basic body diagonal surface given by (1/2/3/1’2’3’ etc). The basic SE equations for this case are as follows.

$\varepsilon_1+\varepsilon_2+\varepsilon_3+\varepsilon_{1'2'3'}=C_{20}$, $\varepsilon_1+\varepsilon_{2'}+\varepsilon_3+\varepsilon_{1'23'}=C_{21}$,
$\varepsilon_{1'}+\varepsilon_{2'}+\varepsilon_3+\varepsilon_{123'}=C_{22}$, $\varepsilon_{1'}+\varepsilon_2+\varepsilon_3+\varepsilon_{12'3'}=C_{23}$.

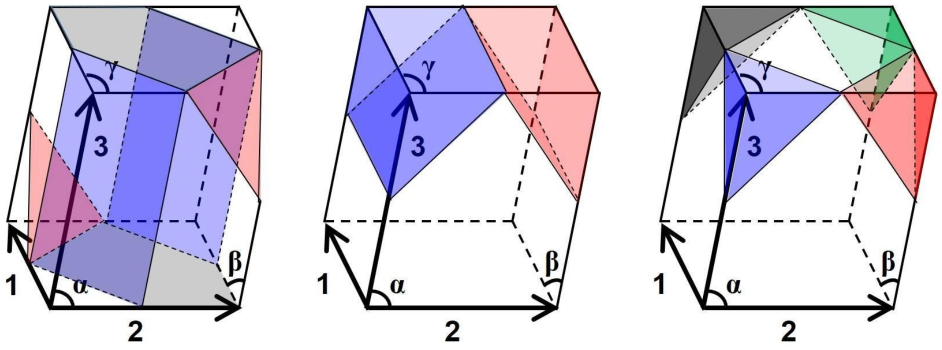

(i) (ii) (iii)

Supplementary Figure 11. In 3D, the solid bodies whose total energies are defined and computable include the slabs as in (i), wedges as examples in (ii), and tetrahedrons in (iii). To count, there are the slabs with

opposite faces 3/3' shaded light grey in (i), similarly 1/1' or 2/2', total 3; then there are two slabs with diagonal faces parallel to an axis like **3**, shaded blue in (i), and similarly two parallel to **1** and two parallel to **2** -- total 6; further the slabs with body-diagonal faces like shaded red in (i), there are total 4 of these. (ii) Shown are two non-equivalent wedges parallel to **1**, plus other pairs of wedges parallel to **2** and to **3** -- total 6. (iii) Non-equivalent tetrahedrons total 4.

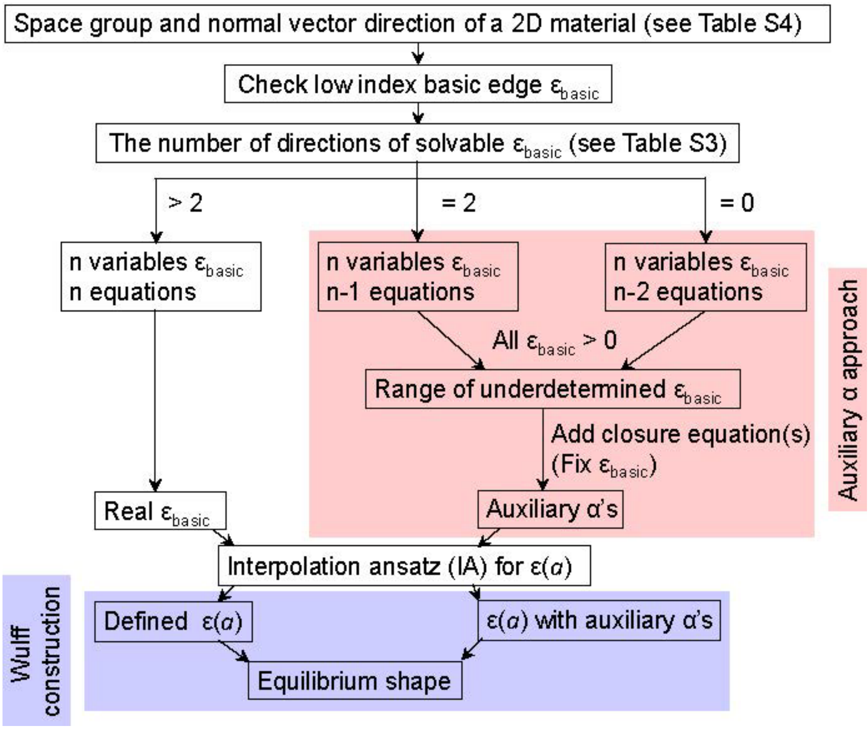

Supplementary Figure 12. The step-by-step flowchart of the determination of equilibrium shape for 2D materials, with "auxiliary energy" closure method enabling the Wulff construction, in Methods.

References:

38. Gibbs, J. W. *The collected works of J. Willard Gibbs*. Vol. 1, p. 219 (Longmans, Green, and Co., New York, 1928).

39. Butt, H., Graf, K. and Kappl, M. *Physics and Chemistry of Interface*s. p. 31 (Wiley-VCH Verlag GmbH & Co. KGaA, Weinheim, 2006)

40. Rowlinson, J. S. and Widom, B. *Molecular Theory of Capillarity*. p. 31 (Oxford Scientific, New York, 1982).

41. Liang, L. *et al.* Electronic Bandgap and Edge Reconstruction in Phosphorene Materials. *Nano Lett.* **14**, 6400–6406 (2014).

42. Ahn, J.-H. *et al.* Deterministic two-dimensional polymorphism growth of hexagonal n-type SnS_2 and orthorhombic p-type SnS crystals. *Nano Lett.* **15**, 3703–3708 (2015).

43. Xia, J. *et al.* Physical vapor deposition synthesis of two-dimensional orthorhombic SnS flakes with strong angle/temperature-dependent Raman responses. *Nanoscale* **8**, 2063–2070 (2016).

44. Mutlu, Z. *et al.* Phase Engineering of 2D Tin Sulfides. *Small* **12**, 2998–3004 (2016).

45. Winterbottom, W. L. Equilibrium shape of a small particle in contact with a foreign substrate. *Acta Metall.* **15**, 303–310 (1967).

46. Kaischew, R. Equilibrium shape and work of formation of crystalline nuclei on a foreign substrate (in Bulgarian). *Commun. Bulg. Acad. Sci.* **1**, 100 (1967).
